# Rapid Identification of Drug Resistance and Phylogeny in M. tuberculosis, Directly from Sputum Samples

**DOI:** 10.1128/spectrum.01252-22

**Published:** 2022-09-14

**Authors:** Martín Barbosa-Amezcua, Betzaida Cuevas-Córdoba, Cristóbal Fresno, Joshua I. Haase-Hernández, Karol Carrillo-Sánchez, Minerva Mata-Rocha, Marcela Muñoz-Torrico, Claudia Bäcker, Vanessa González-Covarrubias, Carmen Alaez-Verson, Xavier Soberón

**Affiliations:** a Laboratorio de Farmacogenómica, Instituto Nacional de Medicina Genómica (INMEGEN), Mexico City, México; b Instituto de Investigaciones Biológicas, Universidad Veracruzana, Xalapa, Veracruz, México; c Departamento de Desarrollo Tecnológico, Instituto Nacional de Medicina Genómica (INMEGEN), Mexico City, México; d Centro de Investigación en Ciencias de la Salud (CICSA), Universidad Anáhuac México, Naucalpan de Juárez, México; e Laboratorio de Diagnóstico Genómico, Instituto Nacional de Medicina Genómica (INMEGEN), Mexico City, México; f Unidad de Investigación Médica en Epidemiología Clínica, UMAE Hospital de Pediatría, Centro Médico Nacional “Siglo XXI”, Instituto Mexicano del Seguro Social (IMSS), Mexico City, México; g Clínica de Tuberculosis y Enfermedades Pleurales, Instituto Nacional de Enfermedades Respiratorias (INER), Mexico City, México; h Laboratorio de Micobacterias, Instituto de Diagnóstico y Referencia Epidemiológicos (InDRE), Secretaría de Salud, Mexico City, México; i Instituto de Biotecnología, Universidad Nacional Autónoma de México (UNAM), Cuernavaca, Morelos, México; Houston Methodist Hospital

**Keywords:** drug resistance, NGS, targeted sequencing, tuberculosis

## Abstract

Tuberculosis (TB) remains one of the most important infectious diseases globally. Establishing a resistance profile from the initial TB diagnosis is a priority. Rapid molecular tests evaluate only the most common genetic variants responsible for resistance to certain drugs, and Whole Genome Sequencing (WGS) needs culture prior to next-generation sequencing (NGS), limiting their clinical value. Targeted sequencing (TS) from clinical samples avoids these drawbacks, providing a signature of genetic markers that can be associated with drug resistance and phylogeny. In this study, a proof-of-concept protocol was developed for detecting genomic variants associated with drug resistance and for the phylogenetic classification of Mycobacterium Tuberculosis (Mtb) in sputum samples. Initially, a set of Mtb reference strains from the WHO were sequenced (WGS and TS). The results from the protocol agreed >95% with WHO reported data and phenotypic drug susceptibility testing (pDST). Lineage genetics results were 100% concordant with those derived from WGS. After that, the TS protocol was applied to sputum samples from TB patients to detect resistance to first- and second-line drugs and derive phylogeny. The accuracy was >90% for all evaluated drugs, except Eto/Pto (77.8%), and 100% were phylogenetically classified. The results indicate that the described protocol, which affords the complete drug resistance profile and phylogeny of Mtb from sputum, could be useful in the clinical area, advancing toward more personalized and more effective treatments in the near future.

**IMPORTANCE** The COVID-19 pandemic negatively affected the progress in accessing essential Tuberculosis (TB) services and reducing the burden of TB disease, resulting in a decreased detection of new cases and increased deaths. Generating molecular diagnostic tests with faster results without losing reliability is considered a priority. Specifically, developing an antimicrobial resistance profile from the initial stages of TB diagnosis is essential to ensure appropriate treatment. Currently available rapid molecular tests evaluate only the most common genetic variants responsible for resistance to certain drugs, limiting their clinical value. In this work, targeted sequencing on sputum samples from TB patients was used to identify Mycobacterium tuberculosis mutations in genes associated with drug resistance and to derive a phylogeny of the infecting strain. This protocol constitutes a proof-of-concept toward the goal of helping clinicians select a timely and appropriate treatment by providing them with actionable information beyond current molecular approaches.

## INTRODUCTION

Tuberculosis (TB) is the 13th global cause of death and, before COVID-19, the first one caused by a single infectious agent. In recent years, the number of new cases has remained stable, with a slight increase in deaths from TB in 2020, despite implementing policies to control it (1).

Although antituberculosis treatments have been available for decades, control of TB has not yet been achieved due, in part, to drug resistance. Drug-resistant tuberculosis (DR-TB) will cause a quarter of the deaths associated with this condition by 2050 (2). In 2019, the WHO (3) reported that 3.4% of new TB cases and 18% of previously treated ones were resistant to rifampicin (RIF) and isoniazid (INH) (MDR), or at least resistant to RIF (RRTB). Additionally, 6.2% of MDR cases were XDR (MDR with additional resistance to at least one fluoroquinolone and one injectable). From 2018 to 2020, only 32% of people diagnosed with MDR/RR-TB received adequate treatment (1).

Establishing a resistance profile through drug susceptibility testing (DST) from the initial TB diagnosis is essential to ensure appropriate treatment, thus reducing the selection and transmission of resistant Mtb, therapeutic failure, and death. Currently, phenotypic DST (pDST) based on bacterial culture is being replaced by genetic tests, such as the Xpert MTB/RIF in its different versions and Line Probe Assays (LiPA), changing the turnaround of drug susceptibility results from several weeks to days or hours (4). However, these molecular methods evaluate only the most common genetic variants responsible for resistance to certain drugs, limiting their clinical value.

The development of rapid and reliable methods for the early determination of DR-TB remains a necessity. Next-generation sequencing (NGS) has the potential to speed up and simultaneously detect all known resistance markers, generating a complete resistance profile, which will contribute to the design of effective treatments from the beginning (5).

Whole Genome Sequencing (WGS) of clinical samples is an expensive and complicated process due to the low bacillary load, which results in high human to bacterial DNA content ratio with consequently low depth of coverage and poor identification of resistance (6). Culture in specialized media for Mtb before NGS ensures sufficient genetic material, but at the expense of limiting the speed at which clinically relevant data are obtained, plus the requirement of biosafety level 3 facilities.

Culture time and expenses can be reduced by sequencing only regions associated with drug resistance (7). Targeted sequencing (TS) panels have shown effectiveness in obtaining resistance profiles directly from clinical samples, avoiding the need for an initial culture, providing a signature of genetic markers associated with drug resistance, and promoting a personalized treatment (8, 9). TS also allows increasing the sequencing depth, facilitating the identification of subpopulations of susceptible and resistant bacteria (heteroresistance), which could impact the early diagnosis of DR-TB (8, 10).

Although the genomes of different members of the Mycobacterium tuberculosis Complex (MTBC) are more than 99.9% identical to each other (11, 12), MTBC strains exhibit important differences in intrinsic resistance to antibiotics, development of drug resistance, pathogenicity, transmissibility, virulence, and the immune response they elicit. These differences that affect the clinical presentation of TB are associated with different phylogenetic lineages (13). Several molecular methods have been used to identify the MTBC, assigning them to lineages (spoligotyping, IS6110-RFLP, MIRU-VNTR, and sequence-based detection of single-nucleotide polymorphisms [SNPs] [14]). SNPs are stable, phylogenetically informative markers that proved capable of differentiating with great discriminatory power the main MTBC lineages and can be easily obtained with an NGS approach (15, 16). Their use for lineage identification is straightforward and can contribute to better clinical management and TB treatment.

Genomic technologies that speed up the diagnosis and simultaneously identify drug-resistant Mtb and lineages will help reduce the spread of resistant strains and achieve better disease control by allowing specific and timely treatment.

This work aimed to develop a proof-of-concept protocol for detecting genomic variants associated with drug resistance and phylogenetic classification of Mtb in sputum samples.

## RESULTS

### Reference strains.

As a first step, the protocol was tested by identifying genetic variants in a set of well-characterized strains. The drug resistance and phylogeny databases were employed to assign them drug resistance profiles and linage classification. The results were compared with the phenotypic results for drug resistance and also with WGS-derived spoligotyping *in silico* for linage.

**(i) Sequencing process.** All reference strains (*n* = 30) were processed, and the DNA recovered was 34.4 ng/μL, on average, which was sufficient for the preparation of TS libraries. Libraries were generated with TruSeq Custom Amplicon and sequenced, obtaining a mean depth of coverage of 2040x and >99% of curated reads mapped to the reference genome.

For the WGS libraries, 3 WHO reference strains and one *H37Rv* showed low DNA concentration and precluded the generation of WGS. The DNA recovered from the 26 strains after library generation with the Nextera XT system was, on average, 9.0 ng/μL. Sequencing afforded, after curating reads, a mean depth of coverage of 63x with >99% of reads mapping to the reference genome.

**(ii) Prediction of drug resistance and phylogeny.** The 28 WHO reference strains comprised only 18 genetically different samples: variants obtained by TS revealed that 10 samples were represented twice. That these were indeed repeated samples (a normal practice with validating panels of the WHO) was confirmed with data derived from WGS. These results were not known in advance by the experimenters because a simple blinded approach was used intentionally to assure the reproducibility of the proposed method. Only the results from 20 reference strains, 18 unique from WHO set, plus two *H37Rv*, are reported in the TS results. Regarding the strains used for WGS, only 17 were available due to the previous exclusion of 3 samples during library generation (16 different WHO strains and one *H37Rv*) and were included for subsequent analysis.

Variants from TS were identified, and the genotypic resistance was determined. In 20 independent samples, statistical indicators of diagnostic tests were calculated for the drugs evaluated. All indicators were 100% for RIF, pyrazinamide (PZA), streptomycin (SM), fluoroquinolones (FQs), and aminoglycosides (AM), showing lower concordance for INH (92.9%) (Table 1). Cohen's kappa coefficient in all drugs showed very good concordance (>0.89) between the results predicted from the genotypic tests and those obtained by pDST. Except for a false negative sample to INH, all identified resistance variants were congruent with the WHO report for these reference strains.

**TABLE 1:** Indicators of the diagnostic test obtained by WGS^*a*^ compared to pDST^*b*^ of Mtb reference strains

Drug	Resistance identified by:		Sensitivity	Specificity	PPV^*c*^	NPV^*d*^	Accuracy	Cohen's Kappa
	Phenotype^*e*^	Genotype^*f*^	(95% CI^*g*^)	(95% CI)	(95% CI)	(95% CI)	(95% CI)	(95% CI)
RIF	13/20	13/20	100 (96.2–100)	100 (92.7–100)	100 (96.2–100)	100 (92.7–100)	100 (97.5–100)	1.0 (1.0–1.0)
INH	14/20	13/20	92.9 (75.8–100)	100 (91.7–100)	100 (96.16–100)	85.7 (52.7–100)	95.0 (83.0–100)	0.89 (0.7–1.0)
PZA	9/20	9/20	100 (94.4–100)	100 (95.5–100)	100 (94.4–100)	100 (95.5–100)	100 (97.5–100)	1.0 (1.0–1.0)
EMB	11/20	11/20	100 (95.5–100)	100 (94.4–100)	100 (95.5–100)	100 (94.4–100)	100 (97.5–100)	1.0 (1.0–1.0)
SM	9/20	9/20	100 (94.4–100)	100 (95.5–100)	100 (94.4–100)	100 (95.5–100)	100 (97.5–100)	1.0 (1.0–1.0)
FQs^*h*^	7/20	7/20	100 (92.9–100)	100 (96.2–100)	100 (92.9–100)	100 (96.2–100)	100 (97.6–100)	1.0 (1.0–1.0)
AM^*i*^	7/20	7/20	100 (92.9–100)	100 (96.2–100)	100 (92.9–100)	100 (96.2–100)	100 (97.6–100)	1.0 (1.0–1.0)
ETH/PTH	3/20	3/20	100 (83.3–100)	100 (97.1–100)	100 (83.3–100)	100 (97.1–100)	100 (97.5–100)	1.0 (1.0–1.0)

a WGS, whole genome sequencing.

b pDST, phenotypic drug susceptibility testing.

c PPV, positive predictive value.

d NPV, negative predictive value.

e Resistant samples identified by pDST/Samples with pDST results.

f Resistant samples identified by sequencing/Samples with sequencing results.

g CI, confidence interval.

h FQs, fluoroquinolones (includes MFX, OFX, and LFX).

i AM, aminoglycosides (includes kanamycin [KAN], amikacin [AMK] and capreomycin [CAP]).

Reads obtained from WGS of 17 Mtb strains were analyzed with the SpoTyping program to get *in silico* spoligotyping results. These results completely agree with the proposed phylogenetic variants set, although *in silico* spoligotyping results, which use the whole genome, had better resolution (Table 2).

**TABLE 2:** Lineage prediction of the Mtb reference strains by TS or WGS^*a*^, validated with SpoTyping

Frequency	Lineage identified by:		
	Targeted-SNPs^*b*^	WGS-SNPs	SpoTyping
1	Wild-type^*c*^	Wild-type	*H37Rv*
2	Haarlem	Haarlem	T1, H1
2	East African Indian	East African Indian	CAS1-Delhi
2	Indo Oceanic	Indo Oceanic	EAI1-SOM, EAI6-BGD1
4	LAM	LAM	LAM1, LAM6, T5-RUS1
5	East Asian	East Asian	Beijing
1	East Asian	East Asian	ND^*d*^
2	Wild-type	Not sequenced by WGS	
1	East Asian	Not sequenced by WGS	

a WGS, whole genome sequencing.

b SNPs, single nucleotide polymorphisms.

c Wild-type, strain without phylogenetic variants (identical to *H37Rv*).

d ND, not determined.

The *H37Rv* strain is considered the wild-type strain by our protocol and, by definition, contained no phylogenetic variants. An East Asian strain was identified with our phylogenetic variants set but not by SpoTyping (ND). Three strains (2 non-duplicated WHO reference strains and one *H37Rv*) with low DNA concentration were not sequenced by WGS (Table 2).

### Clinical samples.

After the initial validation with the reference strains, the protocol and the databases were applied to clinical, sputum samples, in order to provide a proof-of-concept for its prospective use in the clinical area.

**(i) Sequencing process.** The study included 46 clinical samples. Of them, 23 had pDST for first- and second-line drugs by Bactec MGIT 960; 21 had results for first-line drugs only, and 2 were negative controls by smear microscopy and negative-culture (Table S4).

On average, the DNA recovered was 40.8 ng/μL. The TS libraries of 46 sputum samples were obtained with TruSeq Custom Amplicon, and the DNA recovered after libraries generation was an average of 30.5 ng/μL. After sequencing, quality-filtering and mapping to the Mtb reference genome, an average coverage depth of 862x was achieved.

70% of the sputum samples (31 of 44) were helpful for the subsequent analyses, such as variant calling and annotation. In these samples, about 55% of the reads mapped to the Mtb reference genome. However, from the remaining 15 sputum samples (including 2 negative controls), the reads obtained showed poor mapping to *H37Rv* (<10% of reads), resulting in a low depth of coverage (<22x) and a high percentage of lost variants (>20%) during GATK analysis, probably due to low Mtb load. These samples were discarded from further analysis.

**(ii) Prediction of drug resistance.** The genotypic resistance profile for each drug was obtained and compared to the gold standard, pDST results. Table 3 shows a high sensitivity to first-line drugs (>80%), except for SM (50%). Specificity, positive predictive value (PPV), negative predictive value (NPV), and accuracy were high (>70%) for all the drugs analyzed.

**TABLE 3:** Indicators of the diagnostic test obtained by TS compared to pDST^*a*^ of clinical samples

Drug	Resistance identified by:		Sensitivity	Specificity	PPV^*b*^	NPV^*c*^	Accuracy	Cohen's Kappa
	Phenotype^*d*^	Genotype^*e*^	(95% CI^*f*^)	(95% CI)	(95% CI)	(95% CI)	(95% CI)	(95% CI)
RIF	20/31	21/31	100 (97.5–100)	90.9 (69.4–100)	95.2 (83.8–100)	100 (95.0–100–0)	96.8 (88.9–100)	0.93 (0.8–1.0)
INH	12/31	10/31	83.3 (58.0–100)	100 (97.4–100)	100 (95.0–100)	90.5 (75.5–100)	93.6 (83.3–100)	0.86 (0.7–1.0)
PZA	5/31	7/31	100 (90.0–100)	92.3 (80.1–100)	71.4 (30.8–100)	100 (97.9–100)	93.6 (83.3–100)	0.79 (0.5–1.0)
EMB	5/31	8/31	100 (90.0–100)	88.5 (74.3–100)	62.5 (22.7–100)	100 (97.8–100)	90.3 (78.3–100)	0.71 (0.4–1.0)
SM	6/31	3/31	50.0 (1.7–98.3)	100 (98.0–100)	100 (83.3–100)	89.3 (76.0–100)	90.3 (78.3–100)	0.62 (0.2–1.0)
MFX	4/11	3/31	75.0 (20.0–100)	100 (92.9–100)	100 (83.3–100)	87.5 (58.3–100)	90.9 (69.4–100)	0.79 (0.2–1.0)
OFX/LFX	4/18	3/31	75.0 (20.0–100)	100 (96.4–100)	100 (83.3–100)	93.3 (77.4–100)	94.4 (81.0–100)	0.82 (0.5–1.0)
KAN	1/18	0/31	ND^*g*^	ND	ND	ND	ND	ND
AMK	1/18	0/31	ND	ND	ND	ND	ND	ND
CAP	1/11	0/31	ND	ND	ND	ND	ND	ND
ETH/PTH	7/18	3/31	42.8 (0.0–86.7)	100 (95.5–100)	100 (83.3–100)	73.3 (47.6–99.0)	77.8 (55.8–99.8)	0.48 (0.1–0.9)

a pDST, phenotypic drug susceptibility testing.

b PPV, positive predictive value.

c NPV, negative predictive value.

d Resistant samples identified by pDST/Samples with pDST results.

e Resistant samples identified by sequencing/Samples with sequencing results.

f CI, confidence interval.

g ND, not determined.

All clinical samples in which Xpert MTB/RIF identified resistance (*n* = 14) or sensitivity (*n* = 4) were concordant with the TS system and the bioinformatic analysis developed in this study (sensitivity and specificity of 100%).

Cohen’s kappa coefficient showed very good concordance (0.80-1.0) between phenotypic and genotypic results for RIF, INH, and ofloxacin (OFX)/levofloxacin (LFX). Good agreement (0.60-0.79) was identified for PZA, ethambutol (EMB), SM, and moxifloxacin (MFX); and moderate agreement (0.40-0.59) for ethionamide (ETH)/prothionamide (PTH). Concordance for AM was not evaluated due to the absence of genotypically resistant sputum samples.

Table 4 shows the distribution of variants predicted to cause drug resistance observed in the clinical samples analyzed. Variants *rpoB* S450L (S531L), *katG* S315T, *pncA* H57D, *embB* M306I, and *rpsL* K88R were the most frequently found for first-line drugs (RIF, INH, PZA, EMB, and SM, respectively). Two samples with variant *pncA* H57D (a marker for M. bovis) identified in this study were reported as M. bovis by microbiology tests.

**TABLE 4:** Distribution of variants associated with DR-TB observed in clinical samples

Gen	Variants (codon in E. coli^*a*^)	Frequency	Antibiotic
*rpoB*	S450L (S531L)^*b*^	6	Rifampicin
	Q432P (Q513P)	3	
	L452P (L533P)	3	
	H445N (H526N)	2	
	H445L (H526L)	2	
	H445D (H526D)	2	
	D435Y (D516Y)	2	
	D435V (D516V)^*b*^	1	
	V170F (V146F)	1	
*inhA*	c-15t^*c*^	3	Isoniazid/Ethionamide
	I21T	1	Isoniazid
	g-17t^*c*^	1	
*katG*	S315T	5	
	W191R	1	
*pncA*	H57D	2	Pyrazinamide
	H71R	2	
	V163G	1	
	Y41H	1	
	D136N	1	
*embB*	M306I	6	Ethambutol
	G406D	1	
	M306V	1	
*rpsL*	K88R	3	Streptomycin
*gyrA*	A90V	2	Fluoroquinolones (MFX, OFX, LFX)
	D94Y^*d*^^,^^*e*^	2	
	S91P^*d*^^,^^*e*^	2	
	D94G^*d*^	1	
	G88C	1	

a For *rpoB*, codon numbering in parentheses is based on E. coli.

b Variants combined in a single sample resistant to RIF.

c Variants combined in a single sample resistant to INH.

d Variants combined in a single sample resistant to FQs.

e Variants combined in a single sample resistant to FQs.

Five clinical samples were assumed to be false-susceptible, as follows. Four samples were reported as sensitive to RIF by pDST; however, they harbored the disputed variants H445N (*n* = 2) and L452P (*n* = 2) in the *rpoB* gene. Also, one sample was reported as phenotypically sensitive to INH by pDST but it harbored the *katG* S315T mutation. These variants are widely considered true drug resistance markers (17).

Three of the four RIF false-susceptible samples were drug-resistant by Xpert MTB/RIF. The remaining sample only had pDST results available.

**FIG 1:**
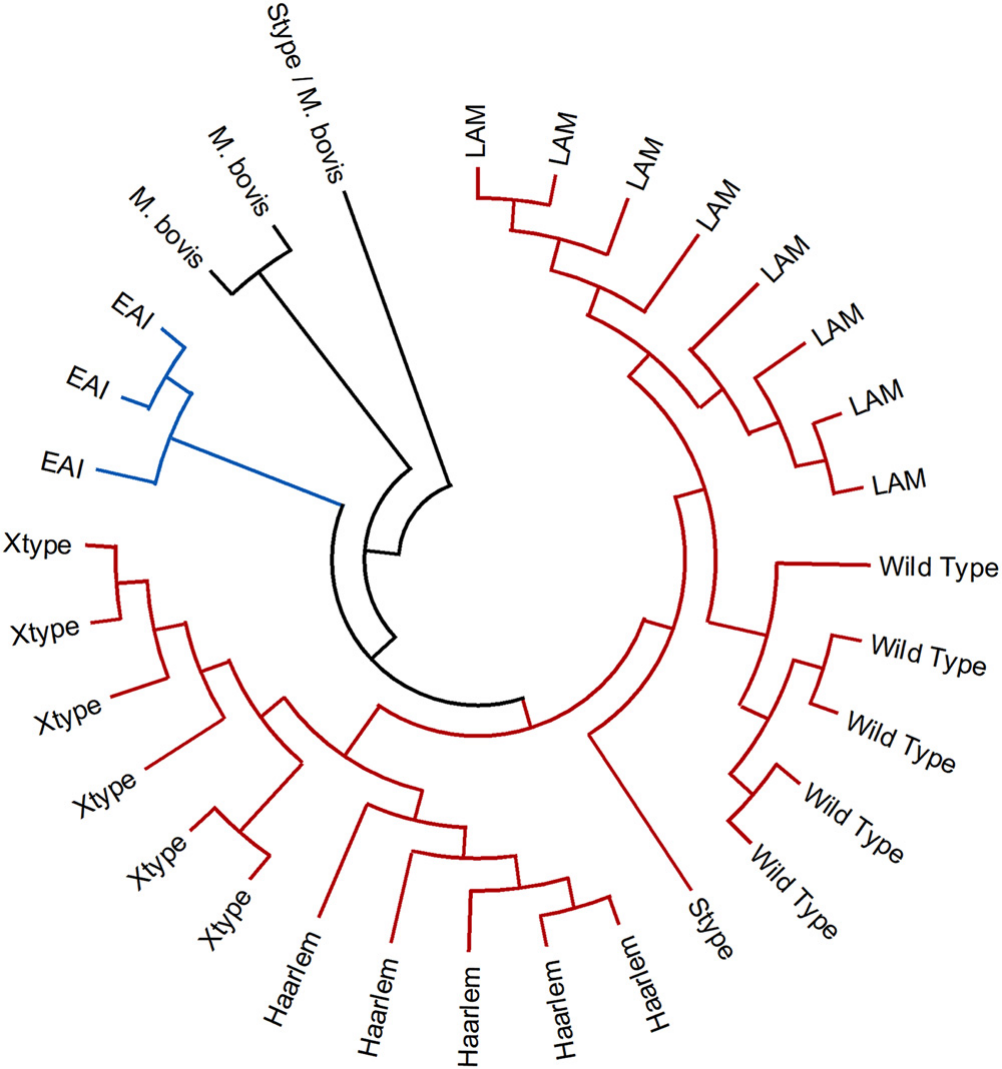
Maximum-likelihood phylogenetic tree of Mtb, obtained by targeted sequencing of clinical samples. Wild-type: Strain without phylogenetic variants (identical to *H37Rv*); Lineage 4 is colored in red; Lineage 3 is colored in blue; M. bovis samples are colored in black; One sample is probably a mixed infection (Stype/M. bovis).

**(iii) Phylogeny prediction.** A maximum-likelihood phylogenetic tree was generated to classify the sputum samples according to their genotype. As expected, the different lineages were grouped (Fig. 1), showing high congruence with our protocol for the phylogenetic variants set.

The presence of 2 lineages of Mtb was observed in sputum samples. The Euro-American lineage was the highest with 20 samples (8 LAM, 6 Xtype, 5 Haarlem, and 1 Stype), followed by the Indo Oceanic with 3. Five samples grouped within the Euro-American lineage by the tree were considered *H37Rv* by our method due to the absence of phylogenetic variants. Variants consistent with M. bovis were found in 2 samples. In 1 sample, variants of M. bovis and the Stype lineage were observed.

### Costs.

Among the factors that need to be considered when developing new diagnostic tests are costs and feasibility. The cost for the test was 287 USD per sample sequenced on a MiSeq (11 samples per run) or 197 USD per sample sequenced on a NextSeq (84 samples per run). The price includes reagents for DNA extraction and quantification (Qubit high sensitivity dsDNA assay), library generation (TruSeq), quality control (Agilent 4200 TapeStation System), and sequencing (MiSeq reagent kit V2 300 cycles or NextSeq Mid Output V2 300 cycle kit).

## DISCUSSION

The incomplete resistance profile of current routine molecular methods such as Xpert MTB/RIF and the Line Probe Assay (LiPA) (18) limits their value for acquiring relevant clinical information. Additionally, different members of the MTBC have been shown to have variations in the clinical phenotype of TB (19) due in part to differences in virulence (20) and the development of drug resistance (21).

Ideally, WGS of Mtb from clinical samples would allow deriving drug resistance and phylogenetic information, but it remains challenging due to the low bacillary load, which complicates the analysis and raises the cost, complicating its use in the clinical context (6, 18). This study proposes a rapid TS protocol, applied to sputum samples, capable of detecting resistance to all first- and some second-line drugs of Mtb and the phylogeny. The results presented show the feasibility of advancing its application as a tool of translational medicine, allowing more effective treatments for TB.

The experimental and bioinformatics pipelines initially tested with Mtb reference strains showed high accuracy (>95%) between the genotype predicted from this protocol and the pDST. Similarly, the reference strain variants reported by the WHO were entirely congruent with those obtained here. Even in clinical samples, the accuracy was >90% for all evaluated drugs, except for ETH/PTH (77.8%). The performance of our genotypic test was similar to other reports (22–25).

Only a few samples disagree between phenotypic and genotypic results. Two clinical samples RIF-susceptible harbored the L452P variant in the *rpoB* gene. This mutation confers a low-level of RIF-resistance (MIC 0.25–1.0 μg/mL) (26, 27) but remains susceptible by pDST. Reducing the critical concentration from 1.0 to 0.5 μg/mL may improve the capacity of phenotypic tests to detect these clinically relevant strains (26).

On the other hand, previous studies have shown that the *rpoB* V170F mutation, located outside the resistance-determining region (RRDR), confers high-level resistance to RIF (MIC >16 μg/mL) (26–28). However, a clinical sample with this mutation was reported as susceptible by pDST. Additional evidence is required to confirm whether this variant is associated with resistance to RIF.

Based on a meta-analysis, Seifert et al. (29) concluded that the mutations in *katG* 315 and *inhA* -15 could explain 83% of resistance to INH. These mutations also were the most common variants in our study with similar sensitivity results (83%).

The evaluation of resistance to PZA, an important drug with sterilizing activity, is complex due to the frequent false positives results obtained by pDST (30, 31). In this study, the sensitivity and specificity to PZA resistance observed in clinical samples (100 and 92%, respectively) were similar to those obtained with WGS from cultures (22, 25).

FQs are part of Group A drugs recommended to treat multidrug-resistant tuberculosis due to their bactericidal effect and good tolerance. Therefore, the WHO (32) recommends, to increase success rates, ruling out FQs resistance before initiating the shorter MDR regimes. Mutations in the quinolone resistance-determining region of *gyrA* and *gyrB* are the main resistance mechanism to FQs (MFX, OFX/LFX). However, these mutations do not fully reflect the complexity of fluoroquinolone resistance (33, 34). Mutations in other genes, including efflux pumps, could explain the observation in this study of a phenotypically resistant sample without mutation in DNA gyrase.

Some resistant samples to SM present variants in the rrs and rpsL genes. Cuevas-Córdoba et al. (35) found mutations in these genes only in 48% of resistant samples to SM, which suggested the presence of additional resistance mechanisms (e.g., *gidB*). Similarly, in this study, mutations in *rrs* or *rpsL* genes were identified in only half of the phenotypically SM-resistant samples.

As reported by other authors with clinical samples and similar sequencing methods (9, 36), the sensitivity was low for ETH/(PTH) (43%). Although only *inhA* gene mutations are currently considered resistance markers for these drugs, the TS panel was designed to capture additional genes related to ETH/PTH resistance (e.g., *ethA*). As with SM, the addition of under-characterized genes or variants to increase the sensitivity to ETH/PTH could lead to a reduction in specificity, potentially excluding useful drugs from the treatment of the disease (17, 37, 38). Therefore, before including poorlys characterized mutations in the analysis, additional studies about their impact on drug resistance are required.

The *eis*, *rrs*, and *tlyA* genes were included in the TS panel to determine resistance to AM. However, sensitivity to AM was not evaluated because the sole phenotypically resistant sample was classified as genotypically sensitive (false negative). Analysis of additional resistance mechanisms is necessary to increase the accuracy, such as the Rv1258c efflux pump (included in this panel), which has AM as substrates (39).

Three discordant samples (phenotypically resistant but genotypically sensitive) for INH and SM showed moderate impact variants in *katG* (G428R) and *gidB* (L16R) genes. Intergenic mutation *inhA* g-17t was observed in one ETH false negative sample. Although these variants could explain the resistance observed in pDST, they are not part of the resistance variants database because they have not been fully characterized. Today, these variants would be considered of “uncertain significance” regarding their role in resistance determination.

Twenty-four variants identified in clinical samples were classified as high-impact (frameshifts, premature stop codons) and 167 as moderate impact variants (missense) by the SnpEff software. These variants were not part of the resistance variants database but could easily be incorporated into future reports when they are better described and validated. Fifteen of these mutations have previously been reported with the potential of generating drug resistance.

In this study, 32% of the clinical samples were MDR, a percentage considerably higher than the 7.94% reported by other authors in México (40). This may be due to the samples' origin in this study, which came from INER, a high specialty hospital, where severe or complicated (e.g., resistant) TB cases are referred.

The sensitivity and specificity for the MDR in clinical samples were 83.3% and 100%, respectively. Within the limits of this study, the sensitivity was only slightly lower than that reported by Madrazo-Moya et al. (23) with WGS from bacterial culture (89%) but with higher specificity (100% versus 97%).

In this study, only 48% (10/21) of the genotypically RIF resistant samples were also INH resistant. Bisimwa et al. (41) reported that only 61.6% of the samples RIF resistant by Xpert MTB/RIF were also INH resistant by MTBDRplus. According to our results, using resistance to RIF as an MDR marker could deprive patients of receiving INH in their treatment, a crucial antituberculosis drug.

Regarding the final terminal efficiency of the method, 31 sputum samples from 44 smear-positive (70%) were successfully processed by TS. Even 2 of the 3 smear-negative and culture-positive samples generated enough data to establish resistance prediction and phylogeny profile. These results are similar to those reported by Doyle et al. (7), who obtained resistance prediction in 74% of the sputum samples analyzed. Feuerriegel et al. (9) and Kayomo et al. (42), using a similar methodology (DeeplexMyc-TB assay), reported a resistance prediction for 78% of the clinical samples.

The presence of heteroresistant variants in Mtb isolates has been reported worldwide for several clinically important drugs (43). However, current molecular methods have limited utility in their detection; Xpert and Xpert Ultra can detect heteroresistance greater than 20% and GenoType greater than 5% (44). Nevertheless, poor detection of heteroresistance can lead to incorrect treatment, amplification of resistance (45), and treatment failure (10).

Detection of minority subpopulations of Mtb (1%) requires a depth of coverage of at least 2000x (10). When sequencing genetically diverse samples, such as sputum, contaminating reads are frequently present (belonging to other microorganisms genetically similar to Mtb), even using TS (46). Based on this consideration, deep sequencing (4000x) was used to ensure that enough reads of Mtb were obtained.

Due to the stringency used to minimize false positive results, approximately 50% of the sequences obtained were quality filtered. After that, contaminating sequences from humans (~3%), viral (0.1%), non-MTB bacteria (~35%), and other microorganisms (~7%) present in the clinical samples were removed; about 55% of high-quality reads mapped to the Mtb reference genome. However, the sequences obtained were sufficient for an adequate phylogenetic and drug resistance determination and to identify heteroresistance in clinical samples.

TS from clinical samples allows heteroresistance identification, improves the phenotype-genotype relation (47), and avoids culture-induced loss of resistant subpopulations (48). In this work, 62% of the variants associated with resistance were fixed (present in ≥95% of the reads [48]), and 38% showed macroheteroresistance (present in 5 to 94% [48]). This method could identify heteroresistance with similar sensitivity compared to current methods.

Interestingly, heteroresistance was observed in FQs-associated variants, even in patients who had not received second-line treatment. Most of these variants are in the fixation process (macroheteroresistance), and samples with 2 or more resistance variants to these drugs were even observed. These data agree with those reported by Zhang et al. (49), who mentioned that this is due to the monotherapeutic use of FQs to treat other infections in patients with undiagnosed TB.

Although WGS is currently the best method used in TB epidemiology (14), it has several drawbacks, as previously discussed. Thus, different studies have focused on SNPs as markers to classify Mtb isolates in their main lineages with a high resolution (15, 16, 50). The TS protocol reported here for sputum samples was able to get similar results to WGS from the bacterial culture at a lower cost.

In this study, a set of 68 SNPs allowed the identification of all MTBC species and Mtb lineages, resulting in a precise method compared with the referenced data. As expected, the Euro-American lineage had the highest presence in the clinical samples, followed by the Indo Oceanic lineage and M. bovis. One sample is probably a mixed infection (Stype/M. bovis). These results coincide with Amlerova et al. (14), who mentions that the LAM sublineage is prevalent in Latin America, with a significant incidence of Xtype and Haarlem in North America and, to a lesser extent, the Indo Oceanic. The prevalence of the Stype sublineage is very low in the world.

Any technological advance must also be evaluated regarding the feasibility of being introduced in the clinical field, considering cost factors and technical practicality for its implementation. In Mexico, the cost of pDST and an XpertMTB/RIF, if not subsidized by international agencies, reaches 250 USD per sample (23). Using our methodology, the cost per sample is 287 USD and could be reduced to 197 USD by sequencing on a NextSeq Mid Output. Considering the constant reduction in sequencing costs and savings derived from processing larger batches, TS of clinical samples could become a viable alternative to obtain effective and timely clinical information, especially in third-level reference hospitals.

Unlike conventional molecular methods, like Xpert MTB/RIF and LiPA, TS and WGS require relatively high computational and laboratory infrastructure (51). This inconvenience could be solved by considering an operation in 2 phases; (i) sample processing and DNA extraction are carried out in first and second-level hospitals, and (ii) the generation of libraries, sequencing, bioinformatic analysis, and reporting of results are carried out in central reference laboratories.

### Limitations.

This study focused on the available information on resistant variants validated microbiologically and genetically. For a better evaluation of the diagnostic method, it will be necessary for future studies to evaluate the performance of this protocol by increasing the number of samples (sensitive and resistant) and including the ability to evaluate discordant results by repeated culture and other molecular tests. Although the panel includes some genes related to resistance to other drugs of clinical importance (linezolid, bedaquiline, and clofazimine), they were not included in the analysis due to the absence of samples with pDST and poor characterization of the variants associated with their resistance. Future studies should add new genes and variants as they are characterized, mainly in drugs with low detection of genotypic resistance. The inclusion of genes such as Mpt64 (31) or hsp65 (52) in the targeted sequencing panel could simplify the identification of Mtb from other nontuberculous mycobacteria (NTM).

Although the spoligotyping results obtained by WGS provided excellent results, having phenotypic lineage assignment for the reference strains and clinical samples would provide double confirmation.

Finally, this proof-of-concept was carried out with a low number of samples; however, it was enough to move toward the next phase involving studies with a greater number of samples with more variability of resistance and phylogeny to validate the TS protocol.

### Conclusions.

Direct sputum sequencing is one attractive option, slightly slower than current molecular methods but with a much better time response than cultures. TS can generate a complete profile of drug resistance, phylogeny, and heteroresistance. Access to this type of molecular information would aid clinicians in selecting the adequate treatment for each person from the beginning of the disease, reducing the transmission of drug resistance between individuals at a manageable cost.

An added advantage of this approach is that, unlike other published methods (e.g., Deeplex Myc-TB), the oligonucleotides in the panel used for TS can be easily synthesized by various suppliers, which provides further opportunities for lowering costs. Furthermore, due to the rapid pace at which new knowledge is generated, with more genes and mutations validated to be associated with drug resistance, these can be readily added to the sequencing panel and the bioinformatics pipeline, increasing the capacity for resistance detection.

## MATERIALS AND METHODS

### Samples.

**(i) Reference strains.** As a first step, well-characterized Mtb reference strains were used to test the protocol and identify drug resistance and phylogeny. Cultures from two *H37Rv* strains and 28 Mtb reference strains were donated by the Instituto de Diagnóstico y Referencia Epidemiológicos (InDRE) from the panel sent annually to Supranational Laboratories by the WHO. The WHO strains are provided with a report with genotypic resistance information; additionally, the InDRE performed pDST for first-line and second-line drugs.

**(ii) Clinical samples (sputum).** In a second stage, the protocol was applied to clinical samples from 46 adult patients collected from February 2014 to February 2016 at the Instituto Nacional de Enfermedades Respiratorias (INER). Forty four of those samples were determined to be TB caused by members of the MTBC. The 2 remaining samples were negative for smear microscopy and culture. Routine tests such as smear microscopy, culture, Xpert MTB/RIF, and pDST were all performed in the Microbiology Laboratory of the INER. An aliquot of the sputum samples (≥500 μL) was inactivated at 80°C for 20 min, transported to Instituto Nacional de Medicina Genómica (INMEGEN), and stored at −80°C until use for the genetic analysis.

### DNA extraction.

DNA extraction from cultures of reference strains and sputum samples was performed following the protocol by Warren et al. (53). The purity of the DNA was evaluated using a NanoDrop One (Thermo Fisher Scientific), the integrity was verified in a 1% agarose gel, and the concentration in a Qubit 3.0 fluorometer (Thermo Fisher Scientific).

### Targeted panel design.

The Illumina Custom Product Design Services was used to design a targeted sequencing panel with 73 genetic regions of Mtb (93 kbp), containing structural genes and regulatory regions, selected from the literature based on their potential association with DR-TB and phylogeny (Table S1). The coordinates of the regions of interest according to the Mtb *H37Rv* genome (NC_000962.3) were sent to Illumina to design and synthesize primers capable of amplifying the full-length of selected genes in a single multiplex PCR assay.

### Libraries generation.

**(i) TS.** TruSeq Custom Amplicon Low Input Kit (Illumina) and the targeted panel described above were used for library generation from reference strains and clinical samples, following the manufacturer's recommendations.

**(ii) WGS.** This was performed on reference strains only. WGS libraries were prepared using the Nextera XT system (Illumina), with 1.0 ng of input DNA, according to the manufacturer's recommendations. To obtain the *in silico* spoligotypes, the sequences obtained were used as input for the SpoTyping software (54); these results were used as the gold standard for linage classification.

### Sequencing.

Sequencing was performed in a NextSeq 500/550, using a Mid Output Kit (2 × 150 bp) (Illumina). A depth of 4000x was estimated for targeted panel libraries (clinical samples and reference strains) to detect minor populations of Mtb in co-infections and possible heteroresistance (10). The depth of WGS libraries (reference strains only) was at least 200x.

The quality of all libraries was evaluated on an Agilent 4200 TapeStation System, and their concentration determined with the Qubit high sensitivity dsDNA assay (Qubit 3.0, Thermo Fisher) before sequencing.

### Bioinformatic analysis.

The bioinformatic analysis of the clinical samples and the strains was carried out following the methodology of Cuevas-Córdoba et al. (46). Briefly, reads were quality-filtered, and human and viral DNA were eliminated. Reads were mapped to the Mtb reference genome NC_000962.3. Specific conditions were applied to identify and discard reads from other microorganisms genetically similar to Mtb (Fig. S1). Reads regarded as true Mtb were used for the variant calling with the GATK software, and the variants were annotated with the SnpEff software.

### Resistance variants and phylogeny databases.

A resistance variants database of 585 mutations (Table S2) associated with phenotypic resistance was created using the variants reported by Miotto et al. (17), WHO (5) and the CRyPTIC Consortium, and the 100,000 Genomes Project (22).

Additionally, a phylogenetic variants database with 68 SNPs, based on the SNPs reported by Coll et al. (15), Lipworth et al. (50), and Feuerriegel et al. (16), was generated. Within the variants present in the genes of the TS panel, those shared by more than 1 lineage were eliminated, until a minimum of 1 and a maximum of 3 SNPs were kept capable of classifying each of the different species and subspecies of the MTBC (Table S3).

### Genotypic-phenotypic correlation of the resistance and phylogeny variants in Mtb.

**(i) Reference strains.** Resistance variants of the reference strains were identified by TS. Genotypic resistance results were assigned to each sample using the resistance variants database and compared with those reported by the WHO and the pDST results (that were considered the gold standard).

WGS of reference strains was also used for *in silico* spoligotyping. Raw reads were used for spoligotyping with the SpoTyping program (54). Results were compared with the lineages predicted by TS, using the phylogenetic variants database generated in this study.

**(ii) Clinical samples.** Resistance variants of the clinical samples were identified by TS. Genotypic resistance was determined for each sample using the resistance variants database. The results were compared with the pDST, which was taken as the gold standard.

The phylogenetic variants were derived from the TS and the lineages were predicted using the phylogenetic variants database.

### Statistical analysis.

The genotypic resistance for each drug, for all samples, was compared with their corresponding pDST. Based on these results, statistical indicators for diagnostic tests such as sensitivity, specificity, positive predictive value (PPV), negative predictive value (NPV), and accuracy for each drug were calculated using the Epidat 3.2 software. The genotype-phenotype agreement was evaluated with Cohen's kappa test using SPSS statistical program.

Clinical samples that were phenotypically susceptible, but harbored mutations associated with drug resistance according to Miotto et al. (17), were assumed to be false-susceptible and considered resistant, disregarding the phenotypic result (5, 17).

### Phylogenetic tree.

The method proposed by Walker et al. (55) was utilized to classify the samples and identify them according to their genotype. Briefly, all the SNPs identified in each clinical sample by TS were concatenated to generate a signature sequence for each sample. Using the Mega X program, a multiple sequence alignment was performed with the signature sequences. A maximum-likelihood phylogenetic tree was inferred by applying the general reversible time model with 1000 bootstraps.

### Ethics.

All participants read and signed an informed consent to participate in this study. All patients' personal information was removed, and the samples were tagged with a code for identification. Both INER and INMEGEN bioethics committees approved the study.

### Data availability.

All relevant data that support the findings described in this study are fully available in GenBank, BioProject accession number PRJNA826553.
