# Supplementary material for: Rapid Identification of Drug Resistance and Phylogeny in M. tuberculosis, Directly from Sputum Samples
Source: Microbiol Spectr. 2022 Sep 14;10(5):e01252-22. doi: 10.1128/spectrum.01252-22 (PMC9602270; doi:10.1128/spectrum.01252-22)
Supplement: Supplemental file 1 — Supplemental material. Download spectrum.01252-22-s0001.pdf, PDF file, 0.4 MB [file spectrum.01252-22-s0001.pdf]

Supplemental Table 1. Structural genes and regulatory regions selected based on their potential association with DR-TB.

Start	End	Locus	Name	Orientation
5240	7267	Rv0005	gyrB	+
7302	9818	Rv0006	gyrA	+
156578	157600	Rv0129c	fbpC	-
157601	157848		Upstream fbpC	-
244484	247318	Rv0206c	mmpL3	-
408449	408633		Upstream Rv0340	+
408634	409173	Rv0340	Rv0340	+
409174	409361		Upstream iniB	+
409362	410801	Rv0341	iniB	+
410838	412760	Rv0342	iniA	+
412757	414238	Rv0343	iniC	+
480355	483231	Rv0402c	mmpL1	-
759807	763325	Rv0667	rpoB	+
763370	767320	Rv0668	rpoC	+
778990	779487	Rv0678	Rv0678	+
781560	781934	Rv0682	rpsL	+
781934	782404	Rv0683	rpsG	+
800809	801462	Rv0701	rplC	+
946056	947315	Rv0849	Rv0849	+
1102542	1105109	Rv0987	Rv0987	+
1406081	1407340	Rv1258c	Rv1258c	-
1416181	1417347	Rv1267c	embR	-
1417348	1417657		Upstream embR	-
1471846	1473382	MTB000019	rrs	+
1473658	1476795	MTB000020	rrl	+
1673300	1673439		Upstream fabG1	+
1673440	1674183	Rv1483	fabG1- mabA	+
1674202	1675011	Rv1484	inhA	+
1792400	1793740	Rv1592c	Rv1592c	-
1793741	1793996		Upstream Rv1592c	-
1833542	1834987	Rv1630	rpsA	+
1917940	1918746	Rv1694	tlyA	+
1929786	1931456	Rv1704c	cycA	-
2006636	2006947	Rv1772	Rv1772	+
2101651	2103042	Rv1854c	ndh	-
2125904	2127967	Rv1877	Rv1877	+
2153889	2156111	Rv1908c	katG	-
2156112	2156148		Upstream katG	-

Supplemental Table 1. Continued

Start	End	Locus	Name	Orientation
2156149	2156592	Rv1909c	furA	-
2284799	2285641	Rv2039c	Rv2039c	-
2288681	2289241	Rv2043c	pncA	-
2289242	2289281		Upstream pncA	-
2516787	2517695	Rv2243	fabD	+
2518115	2519365	Rv2245	kasA	+
2520743	2522164	Rv2247	accD6	+
2714124	2715332	Rv2416c	eis	-
2715333	2715471		Upstream eis	-
2725571	2726087	Rv2427a	OxyR'	-
2726088	2726192		Upstream ahpC	+
2726193	2726780	Rv2428	ahpC	+
2746135	2747598	Rv2447c	folC	-
2869727	2870122	Rv2549c	vapC20	-
3073680	3074471	Rv2764c	thyA	-
3153039	3154631	Rv2846c	efpA	-
3489064	3489505		Upstream moaR1	+
3489506	3490375	Rv3124	moaR1	+
3491808	3492122	Rv3126c	Rv3126c	-
3505284	3505362		Upstream fadE24	+
3505363	3506769	Rv3139	fadE24	+
3644898	3645977	Rv3264c	manB	-
3646895	3647809	Rv3266c	rmlD	-
3647810	3647884		Upstream rmlD	-
3877464	3878507	Rv3457c	rpoA	-
4007331	4008182	Rv3566c	nhoA -nat	-
4043862	4044281	Rv3601c	panD	-
4239863	4243147	Rv3793	embC	+
4243148	4243232		Upstream embA	+
4243233	4246517	Rv3794	embA	+
4246514	4249810	Rv3795	embB	+
4326004	4327473	Rv3854c	ethA	-
4327549	4328199	Rv3855	ethR	+
4407528	4408202	Rv3919c	gidB	-

Supplemental Table 2. Resistance Variants Database.

Gene	Variant	Drug	Annotation	Reference
embB	D328Y	E	Missense	CRyPTIC Consortium, 2018
embB	D354A	E	Missense	CRyPTIC Consortium, 2018
embB	G406A	E	Missense	CRyPTIC Consortium, 2018
embB	G406D	E	Missense	CRyPTIC Consortium, 2018
embB	G406S	E	Missense	CRyPTIC Consortium, 2018
embB	M306I	E	Missense	CRyPTIC Consortium, 2018
embB	M306V	E	Missense	CRyPTIC Consortium, 2018
embB	N1033K	E	Missense	CRyPTIC Consortium, 2018
embB	Q497K	E	Missense	CRyPTIC Consortium, 2018
embB	Q497R	E	Missense	CRyPTIC Consortium, 2018
fabG1	L203L	H	Synonymous	WHO, 2018; CRyPTIC Consortium, 2018
gyrA	A90V	Mfx	Missense	Miotto et al., 2017; WHO, 2018
gyrA	A90V	Ofx/Lfx	Missense	Miotto et al., 2017
gyrA	D89N	Mfx	Missense	WHO, 2018
gyrA	D89N	Ofx/Lfx	Missense	Miotto et al., 2017
gyrA	D94A	Mfx	Missense	Miotto et al., 2017; WHO, 2018
gyrA	D94A	Ofx/Lfx	Missense	Miotto et al., 2017
gyrA	D94G	Mfx	Missense	Miotto et al., 2017; WHO, 2018
gyrA	D94G	Ofx/Lfx	Missense	Miotto et al., 2017
gyrA	D94H	Mfx	Missense	WHO, 2018
gyrA	D94H	Ofx/Lfx	Missense	Miotto et al., 2017
gyrA	D94N	Mfx	Missense	Miotto et al., 2017; WHO, 2018
gyrA	D94N	Ofx/Lfx	Missense	Miotto et al., 2017
gyrA	D94Y	Mfx	Missense	Miotto et al., 2017; WHO, 2018
gyrA	D94Y	Ofx/Lfx	Missense	Miotto et al., 2017
gyrA	G88A	Ofx/Lfx	Missense	Miotto et al., 2017
gyrA	G88C	Mfx	Missense	Miotto et al., 2017; WHO, 2018
gyrA	G88C	Ofx/Lfx	Missense	Miotto et al., 2017
gyrA	S91P	Mfx	Missense	Miotto et al., 2017; WHO, 2018
gyrA	S91P	Ofx/Lfx	Missense	Miotto et al., 2017
gyrB	A504V	Mfx	Missense	WHO, 2018
gyrB	A504V	Ofx/Lfx	Missense	Miotto et al., 2017
gyrB	D461H	Mfx	Missense	WHO, 2018
gyrB	D461N	Mfx	Missense	WHO, 2018
gyrB	E459K	Ofx/Lfx	Missense	Miotto et al., 2017
gyrB	N499D	Mfx	Missense	WHO, 2018

gyrB	N499K	Mfx	Missense	WHO, 2018
gyrB	N499S	Mfx	Missense	WHO, 2018
inhA	I194T	H	Missense	CRyPTIC Consortium, 2018
inhA	I21T	H	Missense	CRyPTIC Consortium, 2018
inhA	S94A	H	Missense	CRyPTIC Consortium, 2018
katG	A109V	H	Missense	CRyPTIC Consortium, 2018
katG	A614E	H	Missense	CRyPTIC Consortium, 2018
katG	D142G	H	Missense	CRyPTIC Consortium, 2018
katG	G125D	H	Missense	CRyPTIC Consortium, 2018
katG	G182R	H	Missense	CRyPTIC Consortium, 2018
katG	G297V	H	Missense	CRyPTIC Consortium, 2018
katG	L141F	H	Missense	CRyPTIC Consortium, 2018
katG	L159P	H	Missense	CRyPTIC Consortium, 2018
katG	L704S	H	Missense	CRyPTIC Consortium, 2018
katG	P232R	H	Missense	CRyPTIC Consortium, 2018
katG	R104Q	H	Missense	CRyPTIC Consortium, 2018
katG	S315I	H	Missense	Miotto et al., 2017; CRyPTIC Consortium, 2018; WHO, 2018
katG	S315N	H	Missense	Miotto et al., 2017; CRyPTIC Consortium, 2018; WHO, 2018
katG	S315T	H	Missense	Miotto et al., 2017; CRyPTIC Consortium, 2018; WHO, 2018
katG	S481L	H	Missense	CRyPTIC Consortium, 2018
katG	S700P	H	Missense	CRyPTIC Consortium, 2018
katG	T180K	H	Missense	CRyPTIC Consortium, 2018
katG	V633A	H	Missense	CRyPTIC Consortium, 2018
katG	W191G	H	Missense	CRyPTIC Consortium, 2018
katG	W191R	H	Missense	CRyPTIC Consortium, 2018
katG	W300C	H	Missense	CRyPTIC Consortium, 2018
katG	W328L	H	Missense	CRyPTIC Consortium, 2018
katG	W505Stop	H	Stop	CRyPTIC Consortium, 2018
katG	W90R	H	Missense	CRyPTIC Consortium, 2018
pncA	A134P	Z	Missense	CRyPTIC Consortium, 2018
pncA	A134V	Z	Missense	Miotto et al., 2017; CRyPTIC Consortium, 2018; WHO, 2018
pncA	A143D	Z	Missense	CRyPTIC Consortium, 2018
pncA	A143G	Z	Missense	WHO, 2018
pncA	A146E	Z	Missense	CRyPTIC Consortium, 2018
pncA	A146S	Z	Missense	CRyPTIC Consortium, 2018
pncA	A146V	Z	Missense	WHO, 2018; CRyPTIC Consortium, 2018
pncA	A152V	Z	Missense	CRyPTIC Consortium, 2018
pncA	A161P	Z	Missense	CRyPTIC Consortium, 2018
pncA	A165D	Z	Missense	CRyPTIC Consortium, 2018
pncA	A170S	Z	Missense	CRyPTIC Consortium, 2018
pncA	A170V	Z	Missense	WHO, 2018; CRyPTIC Consortium, 2018

pncA	A171A	Z	Missense	CRyPTIC Consortium, 2018
pncA	A171E	Z	Missense	Miotto et al., 2017; CRyPTIC Consortium, 2018
pncA	A171T	Z	Missense	WHO, 2018; CRyPTIC Consortium, 2018
pncA	A26V	Z	Missense	CRyPTIC Consortium, 2018
pncA	A28S	Z	Missense	CRyPTIC Consortium, 2018
pncA	A28T	Z	Missense	WHO, 2018
pncA	A30P	Z	Missense	CRyPTIC Consortium, 2018
pncA	A30S	Z	Missense	CRyPTIC Consortium, 2018
pncA	A30V	Z	Missense	CRyPTIC Consortium, 2018
pncA	A36D	Z	Missense	CRyPTIC Consortium, 2018
pncA	A36S	Z	Missense	CRyPTIC Consortium, 2018
pncA	A38A	Z	Missense	CRyPTIC Consortium, 2018
pncA	A38G	Z	Missense	CRyPTIC Consortium, 2018
pncA	A38S	Z	Missense	CRyPTIC Consortium, 2018
pncA	A3A	Z	Missense	CRyPTIC Consortium, 2018
pncA	A3E	Z	Missense	Miotto et al., 2017; CRyPTIC Consortium, 2018; WHO, 2018
pncA	A3P	Z	Missense	WHO, 2018
pncA	A46A	Z	Missense	CRyPTIC Consortium, 2018
pncA	A46E	Z	Missense	CRyPTIC Consortium, 2018
pncA	A46P	Z	Missense	CRyPTIC Consortium, 2018
pncA	A46V	Z	Missense	Miotto et al., 2017; CRyPTIC Consortium, 2018; WHO, 2018
pncA	A79A	Z	Missense	CRyPTIC Consortium, 2018
pncA	A79P	Z	Missense	CRyPTIC Consortium, 2018
pncA	A89V	Z	Missense	CRyPTIC Consortium, 2018
pncA	A92E	Z	Missense	CRyPTIC Consortium, 2018
pncA	C138F	Z	Missense	CRyPTIC Consortium, 2018
pncA	C138G	Z	Missense	CRyPTIC Consortium, 2018
pncA	C138R	Z	Missense	WHO, 2018; CRyPTIC Consortium, 2018
pncA	C138S	Z	Missense	CRyPTIC Consortium, 2018
pncA	C138Stop	Z	Stop	CRyPTIC Consortium, 2018
pncA	C138W	Z	Missense	WHO, 2018
pncA	C138Y	Z	Missense	Miotto et al., 2017; CRyPTIC Consortium, 2018; WHO, 2018
pncA	C14R	Z	Missense	Miotto et al., 2017; CRyPTIC Consortium, 2018; WHO, 2018
pncA	C14Stop	Z	Stop	CRyPTIC Consortium, 2018
pncA	C14W	Z	Missense	CRyPTIC Consortium, 2018
pncA	C14Y	Z	Missense	CRyPTIC Consortium, 2018
pncA	C184R	Z	Missense	CRyPTIC Consortium, 2018
pncA	C72F	Z	Missense	CRyPTIC Consortium, 2018
pncA	C72P	Z	Missense	WHO, 2018

pncA	C72R	Z	Missense	Miotto et al., 2017; CRyPTIC Consortium, 2018; WHO, 2018
pncA	C72Stop	Z	Stop	CRyPTIC Consortium, 2018
pncA	C72W	Z	Missense	CRyPTIC Consortium, 2018
pncA	C72Y	Z	Missense	CRyPTIC Consortium, 2018
pncA	D110E	Z	Missense	CRyPTIC Consortium, 2018
pncA	D110G	Z	Missense	WHO, 2018
pncA	D110H	Z	Missense	CRyPTIC Consortium, 2018
pncA	D110N	Z	Missense	CRyPTIC Consortium, 2018
pncA	D129E	Z	Missense	CRyPTIC Consortium, 2018
pncA	D12A	Z	Missense	Miotto et al., 2017; CRyPTIC Consortium, 2018; WHO, 2018
pncA	D12E	Z	Missense	CRyPTIC Consortium, 2018
pncA	D12G	Z	Missense	Miotto et al., 2017; CRyPTIC Consortium, 2018; WHO, 2018
pncA	D12H	Z	Missense	CRyPTIC Consortium, 2018
pncA	D12N	Z	Missense	Miotto et al., 2017; CRyPTIC Consortium, 2018; WHO, 2018
pncA	D12Y	Z	Missense	CRyPTIC Consortium, 2018
pncA	D136H	Z	Missense	CRyPTIC Consortium, 2018
pncA	D136N	Z	Missense	CRyPTIC Consortium, 2018
pncA	D136V	Z	Missense	CRyPTIC Consortium, 2018
pncA	D136Y	Z	Missense	CRyPTIC Consortium, 2018
pncA	D145E	Z	Missense	CRyPTIC Consortium, 2018
pncA	D158G	Z	Missense	CRyPTIC Consortium, 2018
pncA	D166V	Z	Missense	CRyPTIC Consortium, 2018
pncA	D33A	Z	Missense	WHO, 2018
pncA	D49E	Z	Missense	CRyPTIC Consortium, 2018
pncA	D49G	Z	Missense	Miotto et al., 2017; CRyPTIC Consortium, 2018
pncA	D49H	Z	Missense	CRyPTIC Consortium, 2018
pncA	D49N	Z	Missense	Miotto et al., 2017; CRyPTIC Consortium, 2018
pncA	D49V	Z	Missense	CRyPTIC Consortium, 2018
pncA	D49Y	Z	Missense	CRyPTIC Consortium, 2018
pncA	D56D	Z	Missense	CRyPTIC Consortium, 2018
pncA	D63A	Z	Missense	WHO, 2018
pncA	D63E	Z	Missense	CRyPTIC Consortium, 2018
pncA	D63G	Z	Missense	Miotto et al., 2017; CRyPTIC Consortium, 2018; WHO, 2018
pncA	D63H	Z	Missense	CRyPTIC Consortium, 2018
pncA	D63Y	Z	Missense	CRyPTIC Consortium, 2018
pncA	D80H	Z	Missense	CRyPTIC Consortium, 2018
pncA	D86V	Z	Missense	CRyPTIC Consortium, 2018
pncA	D8A	Z	Missense	WHO, 2018; CRyPTIC Consortium, 2018

pncA	D8E	Z	Missense	Miotto et al., 2017; CRyPTIC Consortium, 2018
pncA	D8G	Z	Missense	Miotto et al., 2017; CRyPTIC Consortium, 2018; WHO, 2018
pncA	D8H	Z	Missense	CRyPTIC Consortium, 2018
pncA	D8N	Z	Missense	Miotto et al., 2017; CRyPTIC Consortium, 2018; WHO, 2018
pncA	D8V	Z	Missense	CRyPTIC Consortium, 2018
pncA	D8Y	Z	Missense	CRyPTIC Consortium, 2018
pncA	E107E	Z	Missense	CRyPTIC Consortium, 2018
pncA	E111Stop	Z	Stop	CRyPTIC Consortium, 2018
pncA	E127D	Z	Missense	CRyPTIC Consortium, 2018
pncA	E127Stop	Z	Stop	CRyPTIC Consortium, 2018
pncA	E144Stop	Z	Stop	CRyPTIC Consortium, 2018
pncA	E15Stop	Z	Stop	CRyPTIC Consortium, 2018
pncA	E173G	Z	Missense	CRyPTIC Consortium, 2018
pncA	E173Stop	Z	Stop	CRyPTIC Consortium, 2018
pncA	E174G	Z	Missense	CRyPTIC Consortium, 2018
pncA	E174K	Z	Missense	CRyPTIC Consortium, 2018
pncA	E181K	Z	Missense	CRyPTIC Consortium, 2018
pncA	E181Stop	Z	Stop	CRyPTIC Consortium, 2018
pncA	E37Stop	Z	Stop	CRyPTIC Consortium, 2018
pncA	E37V	Z	Missense	WHO, 2018
pncA	E91Stop	Z	Stop	CRyPTIC Consortium, 2018
pncA	F106S	Z	Missense	CRyPTIC Consortium, 2018
pncA	F106Y	Z	Missense	CRyPTIC Consortium, 2018
pncA	F13I	Z	Missense	CRyPTIC Consortium, 2018
pncA	F13L	Z	Missense	WHO, 2018
pncA	F13S	Z	Missense	WHO, 2018
pncA	F13V	Z	Missense	CRyPTIC Consortium, 2018
pncA	F13Y	Z	Missense	CRyPTIC Consortium, 2018
pncA	F50F	Z	Missense	CRyPTIC Consortium, 2018
pncA	F58I	Z	Missense	CRyPTIC Consortium, 2018
pncA	F58L	Z	Missense	Miotto et al., 2017; CRyPTIC Consortium, 2018; WHO, 2018
pncA	F58S	Z	Missense	WHO, 2018; CRyPTIC Consortium, 2018
pncA	F81S	Z	Missense	CRyPTIC Consortium, 2018
pncA	F81V	Z	Missense	CRyPTIC Consortium, 2018
pncA	F94C	Z	Missense	WHO, 2018; CRyPTIC Consortium, 2018
pncA	F94F	Z	Missense	CRyPTIC Consortium, 2018
pncA	F94L	Z	Missense	Miotto et al., 2017; CRyPTIC Consortium, 2018; WHO, 2018
pncA	F94S	Z	Missense	Miotto et al., 2017; CRyPTIC Consortium, 2018; WHO, 2018
pncA	G101E	Z	Missense	CRyPTIC Consortium, 2018

pncA	G101Stop	Z	Stop	CRyPTIC Consortium, 2018
pncA	G105D	Z	Missense	WHO, 2018; CRyPTIC Consortium, 2018
pncA	G105R	Z	Missense	CRyPTIC Consortium, 2018
pncA	G105V	Z	Missense	CRyPTIC Consortium, 2018
pncA	G108E	Z	Missense	CRyPTIC Consortium, 2018
pncA	G108R	Z	Missense	Miotto et al., 2017; CRyPTIC Consortium, 2018; WHO, 2018
pncA	G108Stop	Z	Stop	CRyPTIC Consortium, 2018
pncA	G124S	Z	Missense	CRyPTIC Consortium, 2018
pncA	G132A	Z	Missense	Miotto et al., 2017; CRyPTIC Consortium, 2018; WHO, 2018
pncA	G132C	Z	Missense	WHO, 2018; CRyPTIC Consortium, 2018
pncA	G132D	Z	Missense	Miotto et al., 2017; CRyPTIC Consortium, 2018; WHO, 2018
pncA	G132R	Z	Missense	CRyPTIC Consortium, 2018
pncA	G132S	Z	Missense	Miotto et al., 2017; CRyPTIC Consortium, 2018; WHO, 2018
pncA	G132V	Z	Missense	CRyPTIC Consortium, 2018
pncA	G150G	Z	Missense	CRyPTIC Consortium, 2018
pncA	G162A	Z	Missense	CRyPTIC Consortium, 2018
pncA	G162D	Z	Missense	Miotto et al., 2017; CRyPTIC Consortium, 2018
pncA	G162V	Z	Missense	CRyPTIC Consortium, 2018
pncA	G16G	Z	Missense	CRyPTIC Consortium, 2018
pncA	G16R	Z	Missense	CRyPTIC Consortium, 2018
pncA	G16S	Z	Missense	WHO, 2018
pncA	G16V	Z	Missense	CRyPTIC Consortium, 2018
pncA	G17C	Z	Missense	CRyPTIC Consortium, 2018
pncA	G17D	Z	Missense	Miotto et al., 2017; CRyPTIC Consortium, 2018; WHO, 2018
pncA	G23V	Z	Missense	CRyPTIC Consortium, 2018
pncA	G24D	Z	Missense	Miotto et al., 2017; CRyPTIC Consortium, 2018
pncA	G24V	Z	Missense	CRyPTIC Consortium, 2018
pncA	G55C	Z	Missense	CRyPTIC Consortium, 2018
pncA	G75V	Z	Missense	CRyPTIC Consortium, 2018
pncA	G78C	Z	Missense	CRyPTIC Consortium, 2018
pncA	G78D	Z	Missense	CRyPTIC Consortium, 2018
pncA	G78V	Z	Missense	CRyPTIC Consortium, 2018
pncA	G97A	Z	Missense	WHO, 2018
pncA	G97C	Z	Missense	Miotto et al., 2017; CRyPTIC Consortium, 2018
pncA	G97D	Z	Missense	Miotto et al., 2017; CRyPTIC Consortium, 2018; WHO, 2018
pncA	G97R	Z	Missense	CRyPTIC Consortium, 2018

pncA	G97S	Z	Missense	Miotto et al., 2017; CRyPTIC Consortium, 2018; WHO, 2018
pncA	H137D	Z	Missense	WHO, 2018; CRyPTIC Consortium, 2018
pncA	H137P	Z	Missense	Miotto et al., 2017; CRyPTIC Consortium, 2018; WHO, 2018
pncA	H137R	Z	Missense	WHO, 2018; CRyPTIC Consortium, 2018
pncA	H42N	Z	Missense	CRyPTIC Consortium, 2018
pncA	H42Q	Z	Missense	CRyPTIC Consortium, 2018
pncA	H43P	Z	Missense	WHO, 2018; CRyPTIC Consortium, 2018
pncA	H43Q	Z	Missense	CRyPTIC Consortium, 2018
pncA	H51D	Z	Missense	CRyPTIC Consortium, 2018
pncA	H51L	Z	Missense	CRyPTIC Consortium, 2018
pncA	H51N	Z	Missense	CRyPTIC Consortium, 2018
pncA	H51P	Z	Missense	WHO, 2018
pncA	H51Q	Z	Missense	Miotto et al., 2017; CRyPTIC Consortium, 2018; WHO, 2018
pncA	H51R	Z	Missense	Miotto et al., 2017; CRyPTIC Consortium, 2018; WHO, 2018
pncA	H51Y	Z	Missense	WHO, 2018; CRyPTIC Consortium, 2018
pncA	H57D	Z	Missense	Miotto et al., 2017; CRyPTIC Consortium, 2018; WHO, 2018
pncA	H57L	Z	Missense	CRyPTIC Consortium, 2018
pncA	H57N	Z	Missense	WHO, 2018; CRyPTIC Consortium, 2018
pncA	H57P	Z	Missense	Miotto et al., 2017; CRyPTIC Consortium, 2018; WHO, 2018
pncA	H57Q	Z	Missense	WHO, 2018; CRyPTIC Consortium, 2018
pncA	H57R	Z	Missense	Miotto et al., 2017; CRyPTIC Consortium, 2018; WHO, 2018
pncA	H57Y	Z	Missense	Miotto et al., 2017; CRyPTIC Consortium, 2018; WHO, 2018
pncA	H71D	Z	Missense	Miotto et al., 2017; CRyPTIC Consortium, 2018
pncA	H71H	Z	Missense	CRyPTIC Consortium, 2018
pncA	H71L	Z	Missense	CRyPTIC Consortium, 2018
pncA	H71N	Z	Missense	CRyPTIC Consortium, 2018
pncA	H71Q	Z	Missense	Miotto et al., 2017; CRyPTIC Consortium, 2018
pncA	H71R	Z	Missense	Miotto et al., 2017; CRyPTIC Consortium, 2018; WHO, 2018
pncA	H71Y	Z	Missense	Miotto et al., 2017; CRyPTIC Consortium, 2018; WHO, 2018

pncA	H82L	Z	Missense	CRyPTIC Consortium, 2018
pncA	H82Y	Z	Missense	WHO, 2018
pncA	H82R	Z	Missense	Miotto et al., 2017; CRyPTIC Consortium, 2018; WHO, 2018
pncA	I133F	Z	Missense	CRyPTIC Consortium, 2018
pncA	I133N	Z	Missense	WHO, 2018; CRyPTIC Consortium, 2018
pncA	I133S	Z	Missense	WHO, 2018
pncA	I133T	Z	Missense	Miotto et al., 2017; CRyPTIC Consortium, 2018; WHO, 2018
pncA	I31F	Z	Missense	CRyPTIC Consortium, 2018
pncA	I31N	Z	Missense	CRyPTIC Consortium, 2018
pncA	I31T	Z	Missense	CRyPTIC Consortium, 2018
pncA	I52T	Z	Missense	CRyPTIC Consortium, 2018
pncA	I5F	Z	Missense	CRyPTIC Consortium, 2018
pncA	I5M	Z	Missense	CRyPTIC Consortium, 2018
pncA	I5N	Z	Missense	CRyPTIC Consortium, 2018
pncA	I5T	Z	Missense	CRyPTIC Consortium, 2018
pncA	I6F	Z	Missense	CRyPTIC Consortium, 2018
pncA	I6M	Z	Missense	CRyPTIC Consortium, 2018
pncA	I6T	Z	Missense	Miotto et al., 2017; CRyPTIC Consortium, 2018; WHO, 2018
pncA	K48E	Z	Missense	CRyPTIC Consortium, 2018
pncA	K48Stop	Z	Stop	CRyPTIC Consortium, 2018
pncA	K48T	Z	Missense	Miotto et al., 2017; CRyPTIC Consortium, 2018
pncA	K96E	Z	Missense	Miotto et al., 2017; CRyPTIC Consortium, 2018; WHO, 2018
pncA	K96M	Z	Missense	CRyPTIC Consortium, 2018
pncA	K96N	Z	Missense	Miotto et al., 2017; CRyPTIC Consortium, 2018; WHO, 2018
pncA	K96Q	Z	Missense	WHO, 2018; CRyPTIC Consortium, 2018
pncA	K96R	Z	Missense	Miotto et al., 2017; CRyPTIC Consortium, 2018; WHO, 2018
pncA	K96Stop	Z	Stop	CRyPTIC Consortium, 2018
pncA	K96T	Z	Missense	Miotto et al., 2017; CRyPTIC Consortium, 2018; WHO, 2018
pncA	L116P	Z	Missense	Miotto et al., 2017; CRyPTIC Consortium, 2018
pncA	L116Q	Z	Missense	CRyPTIC Consortium, 2018
pncA	L116R	Z	Missense	Miotto et al., 2017; CRyPTIC Consortium, 2018
pncA	L117Q	Z	Missense	CRyPTIC Consortium, 2018
pncA	L120P	Z	Missense	Miotto et al., 2017; CRyPTIC Consortium, 2018; WHO, 2018
pncA	L120Q	Z	Missense	CRyPTIC Consortium, 2018

pncA	L120R	Z	Missense	WHO, 2018
pncA	L151S	Z	Missense	Miotto et al., 2017; CRyPTIC Consortium, 2018
pncA	L151Stop	Z	Stop	CRyPTIC Consortium, 2018
pncA	L156P	Z	Missense	CRyPTIC Consortium, 2018
pncA	L156R	Z	Missense	CRyPTIC Consortium, 2018
pncA	L159P	Z	Missense	Miotto et al., 2017; CRyPTIC Consortium, 2018
pncA	L159Q	Z	Missense	CRyPTIC Consortium, 2018
pncA	L159V	Z	Missense	CRyPTIC Consortium, 2018
pncA	L172P	Z	Missense	Miotto et al., 2017; CRyPTIC Consortium, 2018; WHO, 2018
pncA	L19L	Z	Missense	CRyPTIC Consortium, 2018
pncA	L19P	Z	Missense	Miotto et al., 2017; CRyPTIC Consortium, 2018
pncA	L19Q	Z	Missense	CRyPTIC Consortium, 2018
pncA	L27P	Z	Missense	CRyPTIC Consortium, 2018
pncA	L27Q	Z	Missense	CRyPTIC Consortium, 2018
pncA	L35P	Z	Missense	CRyPTIC Consortium, 2018
pncA	L4F	Z	Missense	CRyPTIC Consortium, 2018
pncA	L4S	Z	Missense	Miotto et al., 2017; CRyPTIC Consortium, 2018; WHO, 2018
pncA	L4Stop	Z	Stop	CRyPTIC Consortium, 2018
pncA	L4V	Z	Missense	CRyPTIC Consortium, 2018
pncA	L4W	Z	Missense	CRyPTIC Consortium, 2018
pncA	L85P	Z	Missense	Miotto et al., 2017; CRyPTIC Consortium, 2018; WHO, 2018
pncA	L85Q	Z	Missense	CRyPTIC Consortium, 2018
pncA	L85R	Z	Missense	Miotto et al., 2017; CRyPTIC Consortium, 2018
pncA	M175I	Z	Missense	Miotto et al., 2017; CRyPTIC Consortium, 2018; WHO, 2018
pncA	M175K	Z	Missense	CRyPTIC Consortium, 2018
pncA	M175T	Z	Missense	Miotto et al., 2017; CRyPTIC Consortium, 2018; WHO, 2018
pncA	M175V	Z	Missense	Miotto et al., 2017; CRyPTIC Consortium, 2018; WHO, 2018
pncA	M1L	Z	Missense	CRyPTIC Consortium, 2018
pncA	N112S	Z	Missense	CRyPTIC Consortium, 2018
pncA	N118N	Z	Missense	CRyPTIC Consortium, 2018
pncA	N118Y	Z	Missense	CRyPTIC Consortium, 2018
pncA	N11T	Z	Missense	CRyPTIC Consortium, 2018
pncA	N149I	Z	Missense	CRyPTIC Consortium, 2018
pncA	N149Y	Z	Missense	CRyPTIC Consortium, 2018
pncA	N188T	Z	Missense	WHO, 2018
pncA	P115A	Z	Missense	CRyPTIC Consortium, 2018
pncA	P115R	Z	Missense	CRyPTIC Consortium, 2018

pncA	P54L	Z	Missense	Miotto et al., 2017; CRyPTIC Consortium, 2018; WHO, 2018
pncA	P54Q	Z	Missense	WHO, 2018; CRyPTIC Consortium, 2018
pncA	P54R	Z	Missense	CRyPTIC Consortium, 2018
pncA	P54S	Z	Missense	Miotto et al., 2017; CRyPTIC Consortium, 2018; WHO, 2018
pncA	P54T	Z	Missense	WHO, 2018; CRyPTIC Consortium, 2018
pncA	P62A	Z	Missense	CRyPTIC Consortium, 2018
pncA	P62L	Z	Missense	Miotto et al., 2017; CRyPTIC Consortium, 2018
pncA	P62P	Z	Missense	CRyPTIC Consortium, 2018
pncA	P62Q	Z	Missense	Miotto et al., 2017; CRyPTIC Consortium, 2018; WHO, 2018
pncA	P62R	Z	Missense	WHO, 2018; CRyPTIC Consortium, 2018
pncA	P62S	Z	Missense	CRyPTIC Consortium, 2018
pncA	P62T	Z	Missense	WHO, 2018
pncA	P69L	Z	Missense	CRyPTIC Consortium, 2018
pncA	P69Q	Z	Missense	CRyPTIC Consortium, 2018
pncA	P69R	Z	Missense	WHO, 2018
pncA	P70A	Z	Missense	CRyPTIC Consortium, 2018
pncA	P70S	Z	Missense	CRyPTIC Consortium, 2018
pncA	P77S	Z	Missense	CRyPTIC Consortium, 2018
pncA	P82Y	Z	Missense	WHO, 2018
pncA	Q10E	Z	Missense	CRyPTIC Consortium, 2018
pncA	Q10H	Z	Missense	CRyPTIC Consortium, 2018
pncA	Q10L	Z	Missense	CRyPTIC Consortium, 2018
pncA	Q10P	Z	Missense	Miotto et al., 2017; CRyPTIC Consortium, 2018; WHO, 2018
pncA	Q10R	Z	Missense	Miotto et al., 2017; CRyPTIC Consortium, 2018; WHO, 2018
pncA	Q10Stop	Z	Stop	CRyPTIC Consortium, 2018
pncA	Q122K	Z	Missense	CRyPTIC Consortium, 2018
pncA	Q122Q	Z	Missense	CRyPTIC Consortium, 2018
pncA	Q122Stop	Z	Stop	CRyPTIC Consortium, 2018
pncA	Q141P	Z	Missense	Miotto et al., 2017; CRyPTIC Consortium, 2018; WHO, 2018
pncA	Q141Stop	Z	Stop	CRyPTIC Consortium, 2018
pncA	R121P	Z	Missense	WHO, 2018
pncA	R123P	Z	Missense	Miotto et al., 2017; CRyPTIC Consortium, 2018; WHO, 2018
pncA	R123S	Z	Missense	CRyPTIC Consortium, 2018
pncA	R140P	Z	Missense	CRyPTIC Consortium, 2018
pncA	R154M	Z	Missense	CRyPTIC Consortium, 2018
pncA	R176P	Z	Missense	CRyPTIC Consortium, 2018

pncA	R29C	Z	Missense	CRyPTIC Consortium, 2018
pncA	S104C	Z	Missense	CRyPTIC Consortium, 2018
pncA	S104G	Z	Missense	CRyPTIC Consortium, 2018
pncA	S104R	Z	Missense	Miotto et al., 2017; CRyPTIC Consortium, 2018; WHO, 2018
pncA	S164P	Z	Missense	WHO, 2018; CRyPTIC Consortium, 2018
pncA	S164S	Z	Missense	CRyPTIC Consortium, 2018
pncA	S164Stop	Z	Stop	CRyPTIC Consortium, 2018
pncA	S164T	Z	Missense	CRyPTIC Consortium, 2018
pncA	S179N	Z	Missense	CRyPTIC Consortium, 2018
pncA	S186F	Z	Missense	CRyPTIC Consortium, 2018
pncA	S18L	Z	Missense	CRyPTIC Consortium, 2018
pncA	S18Stop	Z	Stop	CRyPTIC Consortium, 2018
pncA	S18T	Z	Missense	CRyPTIC Consortium, 2018
pncA	S32I	Z	Missense	CRyPTIC Consortium, 2018
pncA	S32N	Z	Missense	CRyPTIC Consortium, 2018
pncA	S59F	Z	Missense	CRyPTIC Consortium, 2018
pncA	S59P	Z	Missense	Miotto et al., 2017; CRyPTIC Consortium, 2018; WHO, 2018
pncA	S65A	Z	Missense	WHO, 2018
pncA	S65F	Z	Missense	CRyPTIC Consortium, 2018
pncA	S65P	Z	Missense	WHO, 2018
pncA	S66P	Z	Missense	Miotto et al., 2017; CRyPTIC Consortium, 2018
pncA	S66Stop	Z	Stop	CRyPTIC Consortium, 2018
pncA	S67P	Z	Missense	Miotto et al., 2017; CRyPTIC Consortium, 2018; WHO, 2018
pncA	S67Stop	Z	Stop	CRyPTIC Consortium, 2018
pncA	S88Stop	Z	Stop	CRyPTIC Consortium, 2018
pncA	S88T	Z	Missense	CRyPTIC Consortium, 2018
pncA	T100P	Z	Missense	WHO, 2018
pncA	T114A	Z	Missense	CRyPTIC Consortium, 2018
pncA	T114P	Z	Missense	WHO, 2018; CRyPTIC Consortium, 2018
pncA	T114T	Z	Missense	CRyPTIC Consortium, 2018
pncA	T135I	Z	Missense	CRyPTIC Consortium, 2018
pncA	T135N	Z	Missense	Miotto et al., 2017; CRyPTIC Consortium, 2018
pncA	T135P	Z	Missense	Miotto et al., 2017; CRyPTIC Consortium, 2018; WHO, 2018
pncA	T135S	Z	Missense	CRyPTIC Consortium, 2018
pncA	T142A	Z	Missense	Miotto et al., 2017; CRyPTIC Consortium, 2018; WHO, 2018
pncA	T142K	Z	Missense	Miotto et al., 2017; CRyPTIC Consortium, 2018; WHO, 2018

pncA	T142M	Z	Missense	Miotto et al., 2017; CRyPTIC Consortium, 2018; WHO, 2018
pncA	T142P	Z	Missense	WHO, 2018
pncA	T142R	Z	Missense	CRyPTIC Consortium, 2018
pncA	T142T	Z	Missense	CRyPTIC Consortium, 2018
pncA	T153I	Z	Missense	WHO, 2018
pncA	T153N	Z	Missense	WHO, 2018; CRyPTIC Consortium, 2018
pncA	T160K	Z	Missense	CRyPTIC Consortium, 2018
pncA	T160P	Z	Missense	Miotto et al., 2017; CRyPTIC Consortium, 2018
pncA	T160R	Z	Missense	CRyPTIC Consortium, 2018
pncA	T168P	Z	Missense	Miotto et al., 2017; CRyPTIC Consortium, 2018
pncA	T177T	Z	Missense	CRyPTIC Consortium, 2018
pncA	T22I	Z	Missense	CRyPTIC Consortium, 2018
pncA	T47A	Z	Missense	CRyPTIC Consortium, 2018
pncA	T47P	Z	Missense	WHO, 2018
pncA	T47S	Z	Missense	CRyPTIC Consortium, 2018
pncA	T76I	Z	Missense	CRyPTIC Consortium, 2018
pncA	T76P	Z	Missense	Miotto et al., 2017; CRyPTIC Consortium, 2018; WHO, 2018
pncA	T76S	Z	Missense	CRyPTIC Consortium, 2018
pncA	V109L	Z	Missense	CRyPTIC Consortium, 2018
pncA	V125A	Z	Missense	CRyPTIC Consortium, 2018
pncA	V125D	Z	Missense	CRyPTIC Consortium, 2018
pncA	V125F	Z	Missense	Miotto et al., 2017; CRyPTIC Consortium, 2018; WHO, 2018
pncA	V125G	Z	Missense	Miotto et al., 2017; CRyPTIC Consortium, 2018
pncA	V125V	Z	Missense	CRyPTIC Consortium, 2018
pncA	V128A	Z	Missense	CRyPTIC Consortium, 2018
pncA	V128D	Z	Missense	CRyPTIC Consortium, 2018
pncA	V128G	Z	Missense	Miotto et al., 2017; CRyPTIC Consortium, 2018; WHO, 2018
pncA	V130A	Z	Missense	WHO, 2018
pncA	V130E	Z	Missense	CRyPTIC Consortium, 2018
pncA	V130G	Z	Missense	WHO, 2018
pncA	V131D	Z	Missense	CRyPTIC Consortium, 2018
pncA	V131F	Z	Missense	CRyPTIC Consortium, 2018
pncA	V131I	Z	Missense	CRyPTIC Consortium, 2018
pncA	V139A	Z	Missense	Miotto et al., 2017; CRyPTIC Consortium, 2018; WHO, 2018
pncA	V139E	Z	Missense	CRyPTIC Consortium, 2018
pncA	V139G	Z	Missense	Miotto et al., 2017; CRyPTIC Consortium, 2018; WHO, 2018

pncA	V139L	Z	Missense	Miotto et al., 2017; CRyPTIC Consortium, 2018; WHO, 2018
pncA	V139M	Z	Missense	CRyPTIC Consortium, 2018
pncA	V147A	Z	Missense	CRyPTIC Consortium, 2018
pncA	V155E	Z	Missense	WHO, 2018; CRyPTIC Consortium, 2018
pncA	V155G	Z	Missense	Miotto et al., 2017; CRyPTIC Consortium, 2018; WHO, 2018
pncA	V155M	Z	Missense	CRyPTIC Consortium, 2018
pncA	V163A	Z	Missense	WHO, 2018
pncA	V163E	Z	Missense	CRyPTIC Consortium, 2018
pncA	V163G	Z	Missense	WHO, 2018
pncA	V180D	Z	Missense	CRyPTIC Consortium, 2018
pncA	V180F	Z	Missense	Miotto et al., 2017; CRyPTIC Consortium, 2018; WHO, 2018
pncA	V180G	Z	Missense	Miotto et al., 2017; CRyPTIC Consortium, 2018
pncA	V180I	Z	Missense	WHO, 2018
pncA	V183D	Z	Missense	CRyPTIC Consortium, 2018
pncA	V183V	Z	Missense	CRyPTIC Consortium, 2018
pncA	V21A	Z	Missense	CRyPTIC Consortium, 2018
pncA	V21E	Z	Missense	CRyPTIC Consortium, 2018
pncA	V21G	Z	Missense	CRyPTIC Consortium, 2018
pncA	V44D	Z	Missense	CRyPTIC Consortium, 2018
pncA	V7A	Z	Missense	WHO, 2018; CRyPTIC Consortium, 2018
pncA	V7D	Z	Missense	CRyPTIC Consortium, 2018
pncA	V7F	Z	Missense	CRyPTIC Consortium, 2018
pncA	V7G	Z	Missense	Miotto et al., 2017; CRyPTIC Consortium, 2018; WHO, 2018
pncA	V7L	Z	Missense	CRyPTIC Consortium, 2018
pncA	V93E	Z	Missense	CRyPTIC Consortium, 2018
pncA	V93L	Z	Missense	CRyPTIC Consortium, 2018
pncA	V9G	Z	Missense	WHO, 2018
pncA	W119G	Z	Missense	CRyPTIC Consortium, 2018
pncA	W119L	Z	Missense	CRyPTIC Consortium, 2018
pncA	W119R	Z	Missense	CRyPTIC Consortium, 2018
pncA	W119Stop	Z	Stop	CRyPTIC Consortium, 2018
pncA	W68C	Z	Missense	Miotto et al., 2017; CRyPTIC Consortium, 2018; WHO, 2018
pncA	W68G	Z	Missense	Miotto et al., 2017; CRyPTIC Consortium, 2018; WHO, 2018
pncA	W68L	Z	Missense	WHO, 2018; CRyPTIC Consortium, 2018
pncA	W68R	Z	Missense	Miotto et al., 2017; CRyPTIC Consortium, 2018; WHO, 2018
pncA	W68Stop	Z	Stop	CRyPTIC Consortium, 2018

pncA	Y103H	Z	Missense	Miotto et al., 2017; CRyPTIC Consortium, 2018
pncA	Y103Stop	Z	Stop	CRyPTIC Consortium, 2018
pncA	Y34D	Z	Missense	Miotto et al., 2017; CRyPTIC Consortium, 2018; WHO, 2018
pncA	Y34S	Z	Missense	WHO, 2018
pncA	Y34Y	Z	Missense	CRyPTIC Consortium, 2018
pncA	Y41H	Z	Missense	WHO, 2018; CRyPTIC Consortium, 2018
pncA	Y41Stop	Z	Stop	CRyPTIC Consortium, 2018
pncA	Y41Y	Z	Missense	CRyPTIC Consortium, 2018
pncA	Y64Stop	Z	Stop	CRyPTIC Consortium, 2018
pncA	Y95Stop	Z	Stop	CRyPTIC Consortium, 2018
pncA	Y99Stop	Z	Stop	CRyPTIC Consortium, 2018
pncA	R148fs	Z	Indel	Miotto et al., 2017
rpoB	D435A	R	Missense	Miotto et al., 2017; CRyPTIC Consortium, 2018; WHO, 2018
rpoB	D435F	R	Missense	Miotto et al., 2017; CRyPTIC Consortium, 2018; WHO, 2018
rpoB	D435G	R	Missense	Miotto et al., 2017; CRyPTIC Consortium, 2018
rpoB	D435N	R	Missense	Miotto et al., 2017; CRyPTIC Consortium, 2018
rpoB	D435V	R	Missense	Miotto et al., 2017; CRyPTIC Consortium, 2018; WHO, 2018
rpoB	D435Y	R	Missense	Miotto et al., 2017; CRyPTIC Consortium, 2018; WHO, 2018
rpoB	D545E	R	Missense	Miotto et al., 2017; CRyPTIC Consortium, 2018
rpoB	G981D	R	Missense	CRyPTIC Consortium, 2018
rpoB	H445C	R	Missense	Miotto et al., 2017; CRyPTIC Consortium, 2018; WHO, 2018
rpoB	H445D	R	Missense	Miotto et al., 2017; CRyPTIC Consortium, 2018; WHO, 2018
rpoB	H445F	R	Missense	Miotto et al., 2017; CRyPTIC Consortium, 2018
rpoB	H445G	R	Missense	Miotto et al., 2017; CRyPTIC Consortium, 2018; WHO, 2018
rpoB	H445L	R	Missense	Miotto et al., 2017; CRyPTIC Consortium, 2018; WHO, 2018
rpoB	H445N	R	Missense	Miotto et al., 2017; CRyPTIC Consortium, 2018; WHO, 2018
rpoB	H445P	R	Missense	Miotto et al., 2017; CRyPTIC Consortium, 2018; WHO, 2018
rpoB	H445R	R	Missense	Miotto et al., 2017; CRyPTIC Consortium, 2018; WHO, 2018

rpoB	H445Y	R	Missense	Miotto et al., 2017; CRyPTIC Consortium, 2018; WHO, 2018
rpoB	I491F	R	Missense	Miotto et al., 2017; CRyPTIC Consortium, 2018; WHO, 2018
rpoB	L430P	R	Missense	Miotto et al., 2017; CRyPTIC Consortium, 2018; WHO, 2018
rpoB	L452P	R	Missense	Miotto et al., 2017; CRyPTIC Consortium, 2018; WHO, 2018
rpoB	M434I	R	Missense	CRyPTIC Consortium, 2018
rpoB	Q432K	R	Missense	Miotto et al., 2017; CRyPTIC Consortium, 2018; WHO, 2018
rpoB	Q432L	R	Missense	Miotto et al., 2017; CRyPTIC Consortium, 2018; WHO, 2018
rpoB	Q432P	R	Missense	Miotto et al., 2017; CRyPTIC Consortium, 2018; WHO, 2018
rpoB	S431T	R	Missense	Miotto et al., 2017; CRyPTIC Consortium, 2018
rpoB	S441L	R	Missense	Miotto et al., 2017; CRyPTIC Consortium, 2018; WHO, 2018
rpoB	S441Q	R	Missense	Miotto et al., 2017; CRyPTIC Consortium, 2018
rpoB	S450F	R	Missense	Miotto et al., 2017; CRyPTIC Consortium, 2018; WHO, 2018
rpoB	S450L	R	Missense	Miotto et al., 2017; CRyPTIC Consortium, 2018; WHO, 2018
rpoB	S450Q	R	Missense	Miotto et al., 2017; CRyPTIC Consortium, 2018
rpoB	S450W	R	Missense	Miotto et al., 2017; CRyPTIC Consortium, 2018; WHO, 2018
rpoB	S450Y	R	Missense	Miotto et al., 2017; CRyPTIC Consortium, 2018
rpoB	T676P	R	Missense	CRyPTIC Consortium, 2018
rpoB	V170F	R	Missense	CRyPTIC Consortium, 2018
rpoB	V359A	R	Missense	CRyPTIC Consortium, 2018
rpoB	D435fs	R	Indel	Miotto et al., 2017
rpoB	N437fs	R	Indel	Miotto et al., 2017
rpoB	F433fs	R	Dupl	Miotto et al., 2017
rpsL	K43R	S	Missense	Miotto et al., 2017
rpsL	K43T	S	Missense	Miotto et al., 2017
rpsL	K88Q	S	Missense	Miotto et al., 2017
rpsL	K88R	S	Missense	Miotto et al., 2017
rpsL	T40I	S	Missense	Miotto et al., 2017
tlyA	N236K	Cm	Missense	Miotto et al., 2017
ahpC	-57C>T	H	Upstream	CRyPTIC Consortium, 2018
ahpC	-72C>T	H	Upstream	CRyPTIC Consortium, 2018
ahpC	-48G>A	H	Upstream	CRyPTIC Consortium, 2018
eis	-12C>T	Km	Upstream	Miotto et al., 2017

eis	-14C>T	Am	Upstream	WHO, 2018
eis	-14C>T	Km	Upstream	Miotto et al., 2017
eis	-10G>A	Km	Upstream	Miotto et al., 2017
eis	-37G>T	Km	Upstream	Miotto et al., 2017
embA	-12C>T	E	Upstream	CRyPTIC Consortium, 2018
embA	-16C>G	E	Upstream	CRyPTIC Consortium, 2018
embA	-16C>T	E	Upstream	CRyPTIC Consortium, 2018
inhA	-17G>T	H	Upstream	CRyPTIC Consortium, 2018
inhA	-8T>C	H	Upstream	CRyPTIC Consortium, 2018
inhA	-15C>T	Eto/Pto	Upstream	Miotto et al., 2017
inhA	-15C>T	H	Upstream	Miotto et al., 2017; CRyPTIC Consortium, 2018; WHO, 2018
katG	1286_1288delCGC	H	Indel	CRyPTIC Consortium, 2018
katG	1339_1350delCAC GACCTCGTC	H	Indel	CRyPTIC Consortium, 2018
katG	1365delT	H	Indel	CRyPTIC Consortium, 2018
katG	1804_1810delAA CCCGT	H	Indel	CRyPTIC Consortium, 2018
katG	1900_1901insC	H	Indel	CRyPTIC Consortium, 2018
katG	21_22insT	H	Indel	CRyPTIC Consortium, 2018
katG	371delG	H	Indel	CRyPTIC Consortium, 2018
pncA	185_186insA	Z	Indel	CRyPTIC Consortium, 2018
pncA	192_193insA	Z	Indel	CRyPTIC Consortium, 2018
pncA	306_309delGTAC	Z	Indel	CRyPTIC Consortium, 2018
pncA	386_389delATGT	Z	Indel	CRyPTIC Consortium, 2018
pncA	428_429insGCCA GACGGC	Z	Indel	CRyPTIC Consortium, 2018
pncA	456_457insC	Z	Indel	CRyPTIC Consortium, 2018
pncA	463_464insG	Z	Indel	CRyPTIC Consortium, 2018
pncA	470_471insA	Z	Indel	CRyPTIC Consortium, 2018
pncA	48_74delTGGCTC GCTGGCGGTAAC CGGTGGCGC	Z	Indel	CRyPTIC Consortium, 2018
pncA	517_518insG	Z	Indel	CRyPTIC Consortium, 2018
pncA	553_564delAGCT CCTGATGG	Z	Indel	CRyPTIC Consortium, 2018
pncA	-11A>G	Z	Upstream	Miotto et al., 2017; CRyPTIC Consortium, 2018; WHO, 2018
pncA	-12T>C	Z	Upstream	Miotto et al., 2017; CRyPTIC Consortium, 2018
pncA	-7T>C	Z	Upstream	Miotto et al., 2017; CRyPTIC Consortium, 2018; WHO, 2018
rpoB	1278_1286delCA CCAGCCA	R	Indel	CRyPTIC Consortium, 2018
rpoB	1292_1293insC CA	R	Indel	CRyPTIC Consortium, 2018

rpoB	1295_1303delA ATTCATGG	R	Indel	CRyPTIC Consortium, 2018
rpoB	1296_1297insT TC	R	Indel	CRyPTIC Consortium, 2018
rpoB	1299_1304delC ATGGA	R	Indel	CRyPTIC Consortium, 2018
rpoB	1328_1337delT GACCCACAA	R	Indel	CRyPTIC Consortium, 2018
rrs	1473246A>G	Am	Intragenic (rrs)	Miotto et al., 2017
rrs	1473246A>G	Cm	Intragenic (rrs)	Miotto et al., 2017
rrs	1473246A>G	Km	Intragenic (rrs)	Miotto et al., 2017
rrs	1472359A>C	S	Intragenic (rrs)	Miotto et al., 2017
rrs	1472359A>C	S	Intragenic (rrs)	Miotto et al., 2017
rrs	1473247C>T	Am	Intragenic (rrs)	WHO, 2018
rrs	1473247C>T	Cm	Intragenic (rrs)	Miotto et al., 2017
rrs	1473247C>T	Km	Intragenic (rrs)	Miotto et al., 2017
rrs	1472307C>T	S	Intragenic (rrs)	Miotto et al., 2017
rrs	1472358C>T	S	Intragenic (rrs)	Miotto et al., 2017
rrs	1472362C>T	S	Intragenic (rrs)	Miotto et al., 2017
rrs	1473329G>T	Am	Intragenic (rrs)	Miotto et al., 2017
rrs	1473329G>T	Cm	Intragenic (rrs)	Miotto et al., 2017
rrs	1473329G>T	Km	Intragenic (rrs)	Miotto et al., 2017

Supplemental Table 3. Phylogenetic Variants Database

Species/Lineage	Genome Position	Reference	Alternate	Gene
M. tuberculosis sensu stricto				
	6112	G	C	gyrB
Indo Oceanic (lineage 1)	3647591	A	G	rmlD
	8452	C	T	gyrA
Beijing/East Asian (lineage 2)	4243460	C	T	embA
	1834177	A	C	rpsA
Not-Beijing	4246088	A	G	embA
Beijing	4248115	C	T	embB
	4243460	C	T	embA
	4242075	G	A	embC
East African Indian (lineage 3)	3645524	C	T	manB
	762434	T	G	rpoB
Euro-American (lineage 4)				
	2518076	C	T	kasA
Haarlem	760115	C	T	rpoB
	1930028	G	C	cycA
	411371	T	C	iniA
Stype	1105102	A	C	Rv0987
	482418	C	T	mmpL1
Uganda	412017	C	G	iniA
	7539	A	G	gyrA
	3646964	C	G	rmlD
Ural	1793790	A	C	Upstream Rv1592c
	4408213	C	T	Upstream gidB
Xtype	4249408	G	A	embB
	157292	C	T	fbpC
LAM	764995	C	G	rpoC
	1103249	C	T	Rv0987
Cameroon	409296	C	T	Upstream iniB
	4326739	G	C	ethA
	3489938	C	A	moaR1
Ghana	1793851	C	A	Upstream Rv1592c
	2517405	G	A	fabD
Tur	9944	A	C	Upstream gyrA
Mainly T	4249732	C	G	embB
	4240153	G	A	embC
Ethiopian (lineage 7)	412842	A	G	iniC
	1918281	A	C	tlyA

Supplemental Table 3. Continued

Species/Lineage	Genome Position	Reference	Alternate	Gene
M. africanum				
Westafrican 1 (lineage 5)	4244635	T	C	embA
	9566	C	T	gyrA
	2101921	C	T	ndh
Westafrican 2 (lineage 6)	4241843	C	A	embC
	4244379	C	T	embA
M. canetti	4243690	T	C	embA
	4247590	A	G	embB
	4240237	G	C	embC
M. bovis BCG	4247173	G	A	embB
	8624	G	T	gyrA
	2102106	C	G	ndh
M. bovis	9217	A	C	gyrA
	4242970	C	T	embC
	1834859	G	A	rpsA
M. caprae	2714317	C	T	eis
	1673766	C	T	fabG1
	6307	T	G	gyrB
M. microti	5671	C	T	gyrB
	471630	G	C	ndhA
	472502	T	C	ndhA
M. pinnipedii	1674520	C	T	inhA
M. mungi	4242177	C	A	embC
	8134	T	C	gyrA
	1918158	G	A	tlyA
M. orygis	2726378	T	A	ahpC
	4244154	A	G	embA
	5516	A	G	gyrB

Supplemental Table 4. Characteristics of patients included in the study.

	Number
**Sex**	
Male	29
Female	17
**Age**	
20-40	16
41-60	15
>61	5
Unknown	10
**Diabetes**	
Yes	21
No	14
Unknown	11
**Hypertension**	
Yes	2
No	27
Unknown	17
**HIV**	
Yes	1
No	30
Unknown	15
**Phenotypic Drug Susceptivity Test**	
First-Line Only	21
First- and Second-Line	23
Free of TB	2

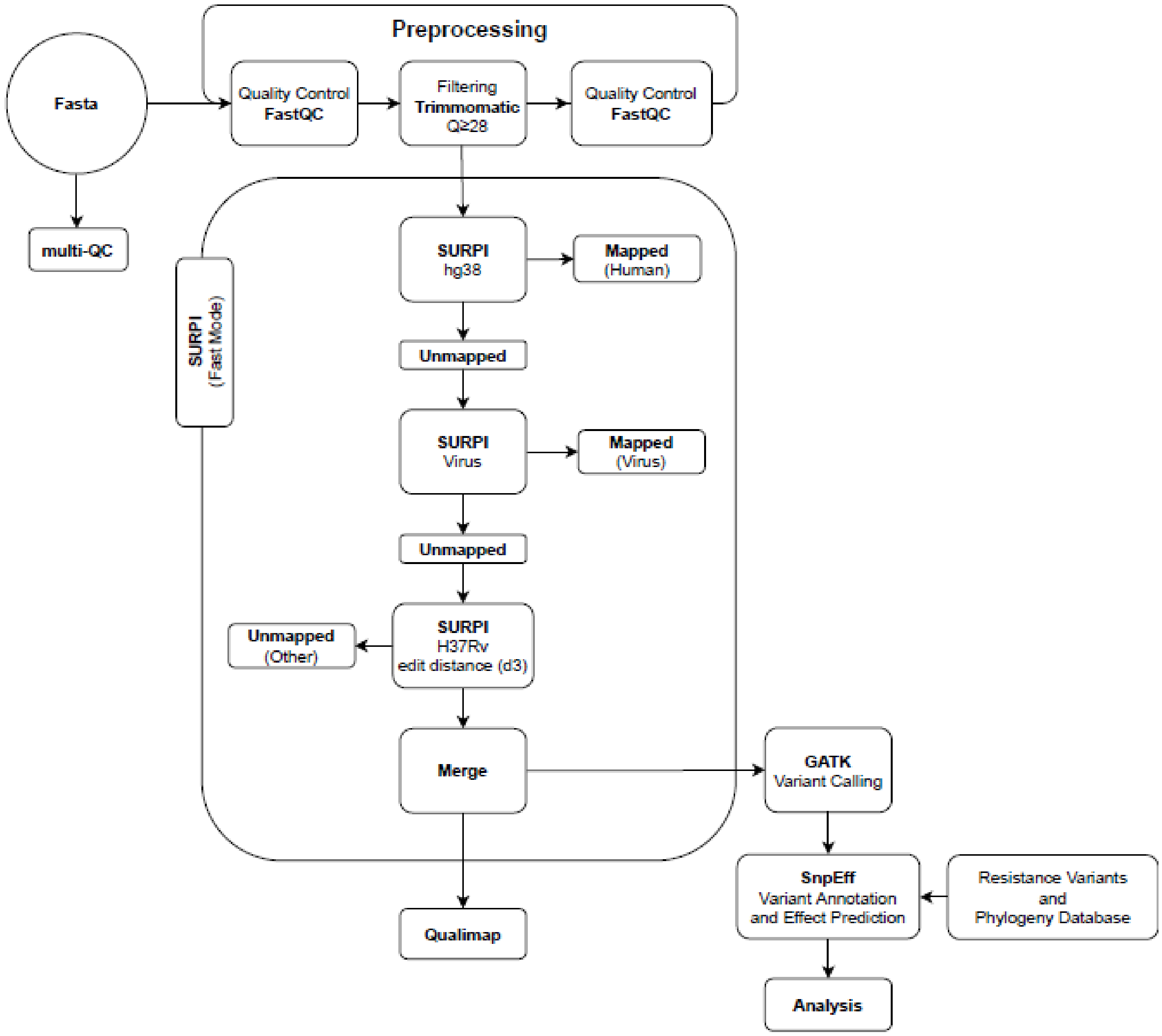

Supplemental Figure 1. Bioinformatic Flowchart
